# Retroviruses use different IP_6_ binding mechanisms to alter the properties of their capsids

**DOI:** 10.1038/s41467-026-76510-7

**Published:** 2026-08-21

**Authors:** J. Ole Klarhof, Donna L. Mallery, James C. V. Stacey, Davide Torre, Michaela Rumlova, Tomas Ruml, John A. G. Briggs, Leo C. James

**Affiliations:** 1 MRC Laboratory of Molecular Biology, Cambridge, UK; 2 Department of Cell and Virus Structure, Max Planck Institute of Biochemistry, Martinsried, Germany; 3 Department of Biotechnology, University of Chemistry and Technology, Prague, Czech Republic; 4 Department of Biochemistry and Microbiology, University of Chemistry and Technology, Prague, Czech Republic

**Keywords:** Microbiology, Cryoelectron microscopy, Cryoelectron microscopy, Retrovirus, Virus structures

## Abstract

HIV-1 uses the metabolite inositol hexakisphosphate (IP_6_) as a host factor to assemble its capsid, but whether this strategy is unique to lentiviruses or represents a common feature of retroviral capsids remains unclear. Here we show that IP_6_ binding is conserved across diverse retroviruses but occurs through distinct capsid sites and mechanisms, and influences viral behaviour. In contrast to HIV-1, the beta-retrovirus Mason-Pfizer Monkey Virus (MPMV) and the gamma-retrovirus Murine Leukaemia Virus (MLV) bind IP_6_ at the threefold lattice interface between capsomers rather than within capsomer pores. Cryo-EM structures of core-like particles reveal that two lysine residues from each capsomer coordinate IP_6_ between either two discrete three-lysine rings (MPMV) or a single heterogeneous six-lysine ring (MLV). MPMV and MLV are largely insensitive to IP_6_ availability in producer cells, but this binding mode renders them highly dependent on IP6 in target cells – the opposite of the dependency pattern of HIV-1. The way in which retroviruses use IP_6_ to build their capsids alters their dependence on the metabolite at different stages of the replicative cycle and in key capsid behaviours, such as assembly and stability.

## Abstract (web summary)

Diverse retroviruses conserve IP_6_ as a molecular glue for core assembly but use it differently: HIV-1 binds IP_6_ within capsomer pores and needs it during virus production, while MPMV and MLV bind IP_6_ between capsomers and require it during infection.

## Introduction

Retroviruses are a class of RNA viruses with a unique ability to reverse transcribe their RNA genome into DNA and integrate it into the host cell’s genome. Successful gene delivery is orchestrated by the viral capsid, a multifunctional transport vehicle, reaction chamber and protective shell. Upon viral fusion with the target cell, the mature core is immediately released into the cytosol, where it must maintain its integrity for hours in order to sequester and protect the viral genome^1,2^, act as a reaction vessel for DNA synthesis^3,4^, mediate intracellular transport and nuclear entry^5,6^, and uncoat at exactly the right time and place^7^. Ligands or mutations that make the capsid too stable or too unstable result in lost infectivity due to improper genome processing, immune detection, or failure to integrate^8,9^.

Understanding how capsid cores self-assemble, function, and disassemble is thus critical for understanding infection and guiding the development of new antivirals. Moreover, as capsids are sophisticated nanoparticles that can be repurposed for cell delivery, there is much to learn from studying them.

The most intensely studied retrovirus is HIV-1, which assembles its capsids over a two-step process. First, it forms an immature lattice comprised of Gag and Gag-Pol polyproteins that package the viral RNA. Second, these polyproteins are cleaved to release capsid protein (CA) for core assembly inside the virion. The HIV-1 mature capsid comprises around 250 hexameric and exactly 12 pentameric capsomers^10^. These hexamers and pentamers are in turn formed around one or two molecules of the strongly negatively charged host metabolite inositol-hexakisphosphate (IP_6_) - an essential building block for the core^3,11^.

During HIV-1 assembly in the producer cell, IP_6_ is actively packaged into the virion by the immature Gag lattice and is thereby available at high concentration during capsid processing^12^. Poly-anionic IP_6_ is coordinated in the central pore of every mature capsomer; HIV-1 has two rings formed by Arg-18 and Lys-25 that can each bind one IP_6_ molecule. This interaction is essential and sufficient to stimulate the formation of core-like particles (CLPs) in vitro^11,13–15^. Equally important, IP_6_ prolongs the mature core’s integrity from minutes to many hours^3,16,17^.

The critical importance of IP_6_ in HIV-1 capsid assembly and stability is illustrated by experiments that alter IP_6_ levels in cells or mutate capsid residues. Removing the kinases responsible for IP_6_ synthesis in producer cells results in reduced virion production, processing defects, defective capsid morphology, reduced capsid stability and loss of infectivity^12,18–20^. Mutating the lysines in the immature lattice responsible for IP_6_ packaging (K158 and K227) broadly phenocopies kinase knockout, with the exception that immature lattice assembly and virion production of the mutants become IP_6_-independent. This illustrates that IP_6_ is only essential for the mature capsid^12^. The role of IP_6_ in the target cell, i.e. post-entry, is less clear. In vitro experiments clearly show that without IP_6_ the capsid rapidly collapses, yet depletion of IP_6_ in target cells has almost no impact on infectivity^19^, unless combined with capsid hypostability mutants^21^. Unlike target cell IP_6_ manipulation, mutating the IP_6_ coordinating residues in the mature capsid (R18 or K25) has a catastrophic effect on viral fitness^22^; likely a combination of impaired assembly and stability, because R18 and K25 are responsible for importing nucleotides into the core for DNA synthesis^4^.

All retroviruses build and maintain a capsid, yet whether IP_6_ is important outside of the lentiviral genus is largely unknown. None of the other five extant orthoretrovirinae genera has a reported IP_6_ binding pocket in the immature lattice like HIV-1. Immature lattice structures of gamma-retrovirus Murine Leukaemia Virus (MLV), or delta-retrovirus Human T-cell Leukaemia Virus type 1 (HTLV-1) did not identify any bound IP_6_ within the resolved parts of the Gag structure^23,24^. In vitro experiments of RSV immature core assembly showed no requirement for IP_6_^25^. Existing cellular data are conflicting, with some reports suggesting modest production defects of RSV and MLV in IPPK knockout (reduced IP_6_ concentration) cells^18^, whilst others reported no effect on MLV^26^.

Compared with those of immature capsids, the central pores of mature capsomers show greater structural similarity across orthoretrovirinae. While the exact arrangement of R18 and K25 found in HIV-1 is not conserved - indeed is not preserved even amongst lentiviruses – the gamma-retrovirus genus, including MLV, is the only one that does not have at least one charged residue in helix 1 that lines the pore (Fig. S1). However, empirical data supporting IP_6_ binding outside of the lentiviral genus is limited. In cryoET data of the alpha-retrovirus RSV, there is density consistent with IP_6_ binding near K17 and R21 - residues positioned similarly to those in the central capsomer pore of HIV-1^25^. Conversely, IP_6_ was not observed in a structure of the HTLV-1 CA N-terminal domain (NTD) solved from crystals soaked in the metabolite^27^, and only a faint density in this position of the immature capsid was observed by CryoEM^28^, despite featuring a lysine ring (K18) positioned equivalently to the HIV-1 R18 and the RSV K17 rings. The beta-retrovirus Mason-Pfizer Monkey Virus (MPMV) lacks a direct equivalent of R18 or K25 yet contains a lysine residue one helical turn below R18 (equivalent to K21 in RSV) that could serve as a binding site for IP_6_ (Fig. S1). In contrast, the epsilon-retrovirus Walleye Dermal Sarcoma Virus (WDSV) features a charged ring at the position corresponding to K25 in HIV-1. At present, there is no structural data available to assess IP_6_ binding in either beta- or epsilon-retroviruses.

Cellular data in support of a role for IP_6_ in the mature capsid across retroviruses are also inconclusive. Depletion of IP_6_ in producer cells reduces the infectious titre of RSV, likely due to defects in immature Gag lattice assembly and budding. Whether IP_6_ has additional effects on maturation or post-entry capsid instability is unknown^25^. MLV, a gamma-retrovirus and hence without a charged residue in helix 1, was reported to have reduced infectivity in cells depleted of IP_5_ and IP_6_, suggesting either of these is required for infectivity^26^. However, the modest phenotypes of MLV in IP-depleted producer and target cells are inconsistent with the complete loss of infectivity when the residue proposed to bind IP_6_, R3, is mutated.

In this study, we have taken a combined biochemical, virological, and structural approach to investigate whether IP_6_ is a conserved cofactor across diverse retroviruses, focusing on the beta-retrovirus MPMV and the gamma-retrovirus MLV.

## Results

### MPMV binds IP_6_ for mature core assembly but not in the central pore

**Fig. 1:**
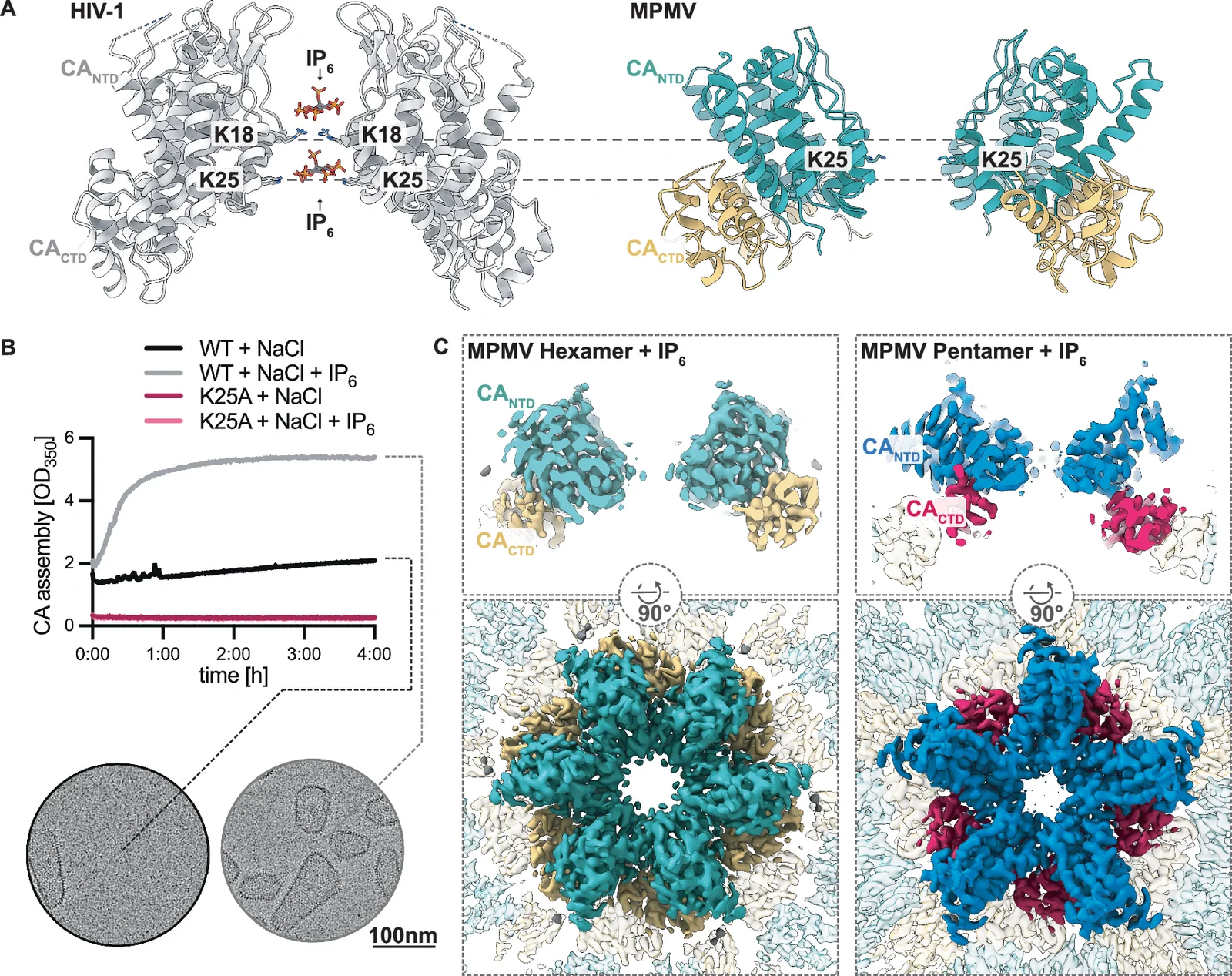
MPMV binds IP_6_ for mature core assembly but not in the central pore. **A** Comparison of the HIV-1 hexamer pore (left, 8CKV, EMD-16703) with the MPMV hexamer pore (right, this study). HIV-1 contains two charged rings, each of which coordinates one IP_6_ molecule. MPMV features an analogous lysine ring positioned to potentially bind one IP_6_ molecule. **B** Assembly reaction kinetics of MPMV capsid for MPMV WT capsid protein (880 µM) and MPMV K25A capsid protein (880 µM), monitored by optical density at *λ* = 350 nm (OD_350_). Reactions were carried out in 100 mM NaCl with or without 1 mM IP_6_, demonstrating IP_6_-driven assembly of WT capsid but not the K25A mutant. The shown reaction is representative of three independent replicates. Representative micrographs depict core assembly in equivalent conditions. **C** Single-particle reconstructions of the MPMV hexamer (CA_NTD_: teal, CA_CTD_: yellow) and pentamer (CA_NTD_: blue, CA_CTD_: red) reveal the absence of a pore density. The surface representations are shown from an outside perspective and as a cross-section.

Most lentiviruses and diverse retroviruses, including RSV and HTLV-1, preserve a charged residue in mature capsomers at a structurally equivalent position to HIV-1 R18 (Fig. S1A, B). MPMV lacks a charged residue here but does have a lysine one turn below the helix equivalent to N21 in HIV-1 (but numbered K25 in MPMV). As MPMV K25 occupies a pore-facing position, the current understanding suggests it would be a strong candidate for IP_6_ binding (Fig. 1A). In HIV-1, IP_6_ coordination is sufficient to mediate spontaneous assembly of core-like particles (CLPs)^11^. We therefore tested whether IP_6_ could similarly induce MPMV CLP formation by exposure of purified MPMV protein to different buffer conditions. MPMV CA partially assembled into cores at a concentration of 880 µM in the absence of IP_6_ (Fig. 1B). However, adding 1 mM IP_6_ substantially increased assembly efficacy, as substantiated by a higher final optical density at *λ* = 350 nm (OD_350_), establishing that MPMV can use IP_6_ as an assembly factor. IP_6_-driven assembly was lost upon mutation of K25 to alanine, suggesting that MPMV K25 is functionally equivalent to HIV-1 R18 (Fig. 1B). To provide direct evidence for this interaction, we determined the cryoEM structure of MPMV hexamers (3.2 Å) and pentamers (3.3 Å) from CLPs in the presence of 5 mM IP_6_, applying a single-particle electron microscopy pipeline^29–31^.

Surprisingly, we were unable to detect any density consistent with IP_6_ adjacent to K25, or indeed any position within the central pore of hexamers and pentamers (Fig. 1C). This indicates that IP_6_ is not bound in the pore despite the fact that CLPs were assembled in the presence of 5 mM IP_6_.

### The MPMV pore residue K25 forms a salt bridge with E26, which is essential for infectivity

**Fig. 2:**
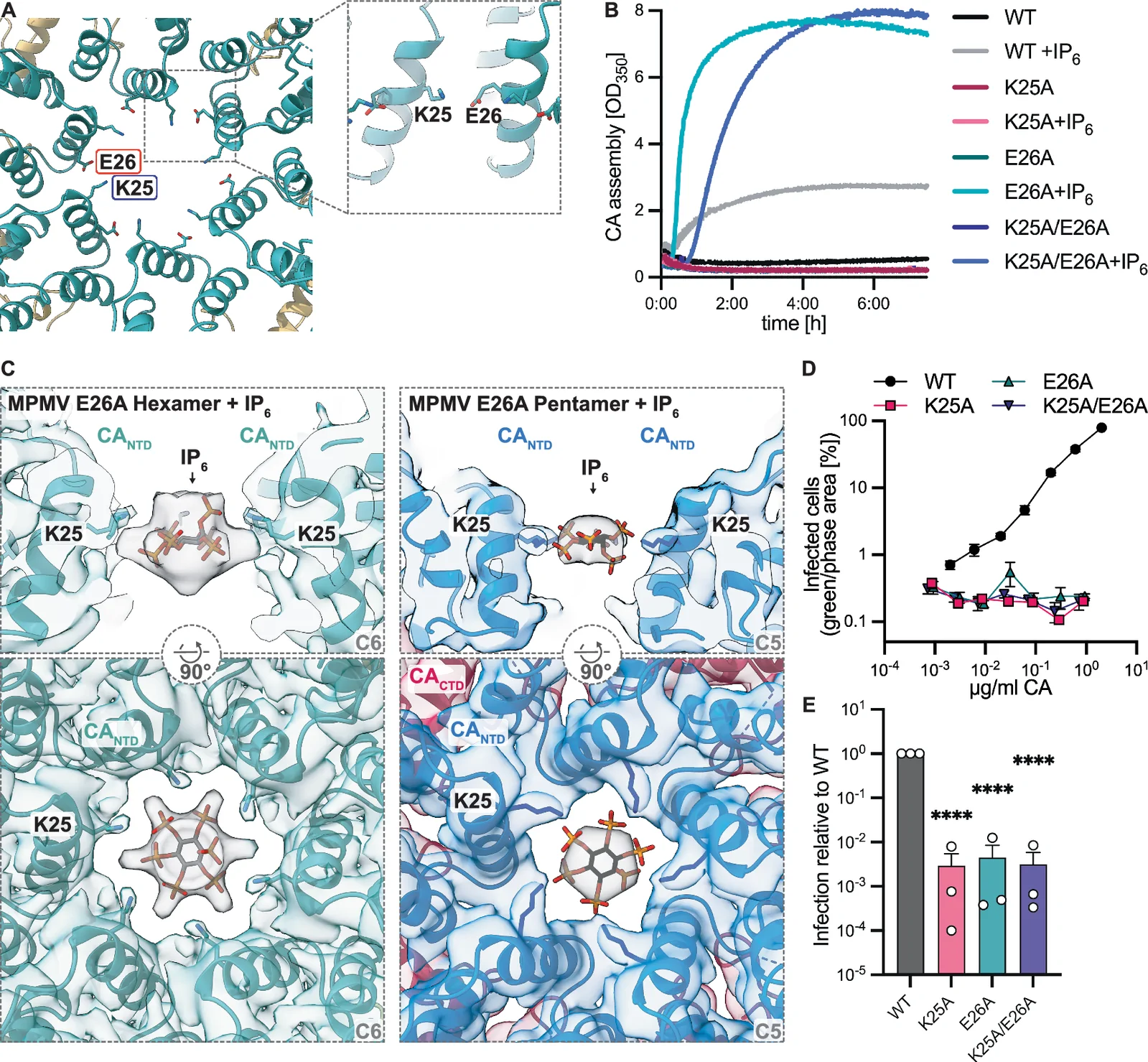
The MPMV pore residue K25 forms a salt bridge with E26, which is essential for infectivity. **A** Model of the MPMV capsomer pore. The relative positions of K25 and E26 suggest their capacity to form an inter-monomer salt bridge. **B** The salt-bridging residues were mutated individually and in combination. The mutant’s assembly capacity was measured at 500 µM MPMV CA protein concentration in the absence or presence of 1 mM IP_6_. **C** The cryoEM structure of the MPMV E26A capsid reconstruction with 5 mM IP_6_ shows the central capsomer pore from the outside and in cross-section. The surface representation and corresponding atomic models are depicted for the hexamer (CA_NTD_: teal, CA_CTD_: yellow) and pentamer (CA_NTD_: blue, CA_CTD_: red). An IP_6_ molecule was placed into the central pore density, demonstrating its coordination by K25 in the absence of E26. **D** Representative infections with indicated viruses are plotted as the infected percentage of the total cell area against the quantity of input virus. **E** The bar chart summarises the infectivity of mutant viruses relative to WT from three independent experiments. Mutations of the salt-bridging residues in the pore abolish infectivity, yielding non-infectious virions. A one-sample two-tailed *t*-test was used to compare the mean of a mutant sample against the hypothetical mean of 1. Data are presented as mean values ± SEM. Significant differences are indicated (ns *p* > 0.05, * *p* ≤ 0.05, ** *p* ≤ 0.01, *** *p* ≤ 0.001, **** *p* ≤ 0.0001, *n* = 3 biological replicates).

To understand why K25A had disrupted IP_6_-driven CLP formation despite apparently not binding the metabolite, we examined the residues surrounding K25 in the assembled lattice reconstruction. This revealed that within capsomers, a glutamic acid (E26) from one monomer is positioned to form a salt bridge with K25 of a neighbouring monomer (Fig. 2A). Based on this observation, we hypothesised that K25 could stabilise the capsomer by forming a salt bridge with E26 or that it could have a dual role, dynamically engaging in both IP_6_ coordination and E26 binding.

To discriminate between these hypotheses, we generated a panel of mutants and assessed IP_6_-driven CLP assembly. In contrast to K25A, E26A capsid could be assembled in the presence of IP_6_ (Fig. 2B). We solved the structure of E26A MPMV CLPs assembled in vitro under the same conditions as the wild-type reconstruction (5 mM IP_6_). In contrast to WT, E26A hexamers and pentamers bound IP_6_ in the central capsomer pore, coordinated by the concentric K25 ring (Fig. 2C). Thus, the difference in K25A and E26A assembly can be explained because whilst mutation of either residue breaks the salt-bridge, K25A leaves the uncompensated, disruptive negative charge from E26 at the centre of the pore. In the E26A mutant, however, the charge of K25 can be compensated by IP_6_.

However, this does not explain why no IP_6_ was seen at the pore in WT CLPs despite its IP_6_-driven assembly. We therefore asked how assembly is altered by simultaneous K25A/E26A mutation. Strikingly, the double mutant was able to assemble in the presence of IP_6_ and with greater efficacy than WT (Fig. 2B). To understand the importance of these salt bridge residues to viral fitness we examined the impact of their mutation on viral production and infection. While neither K25A, E26A, nor the double mutant substantially reduced viral production (Fig. S2A), all mutations were effectively non-infectious (Fig. 2D, E). This suggests that the K25/E26 salt bridge, though not essential for in vitro assembly, is required for infectivity. It may play a role in maintaining capsid stability, or possibly K25 is used to mediate dNTP import, similar to R18 and K25 in HIV-1^4^, and interaction with E26 occurs in the absence of nucleotides to prevent charge repulsion. Importantly, the data show that IP_6_ must be driving MPMV assembly through an unknown interface elsewhere in the capsid lattice and not in the central pore.

### IP_6_ is bound at the threefold axis of the MPMV capsid lattice

**Fig. 3:**
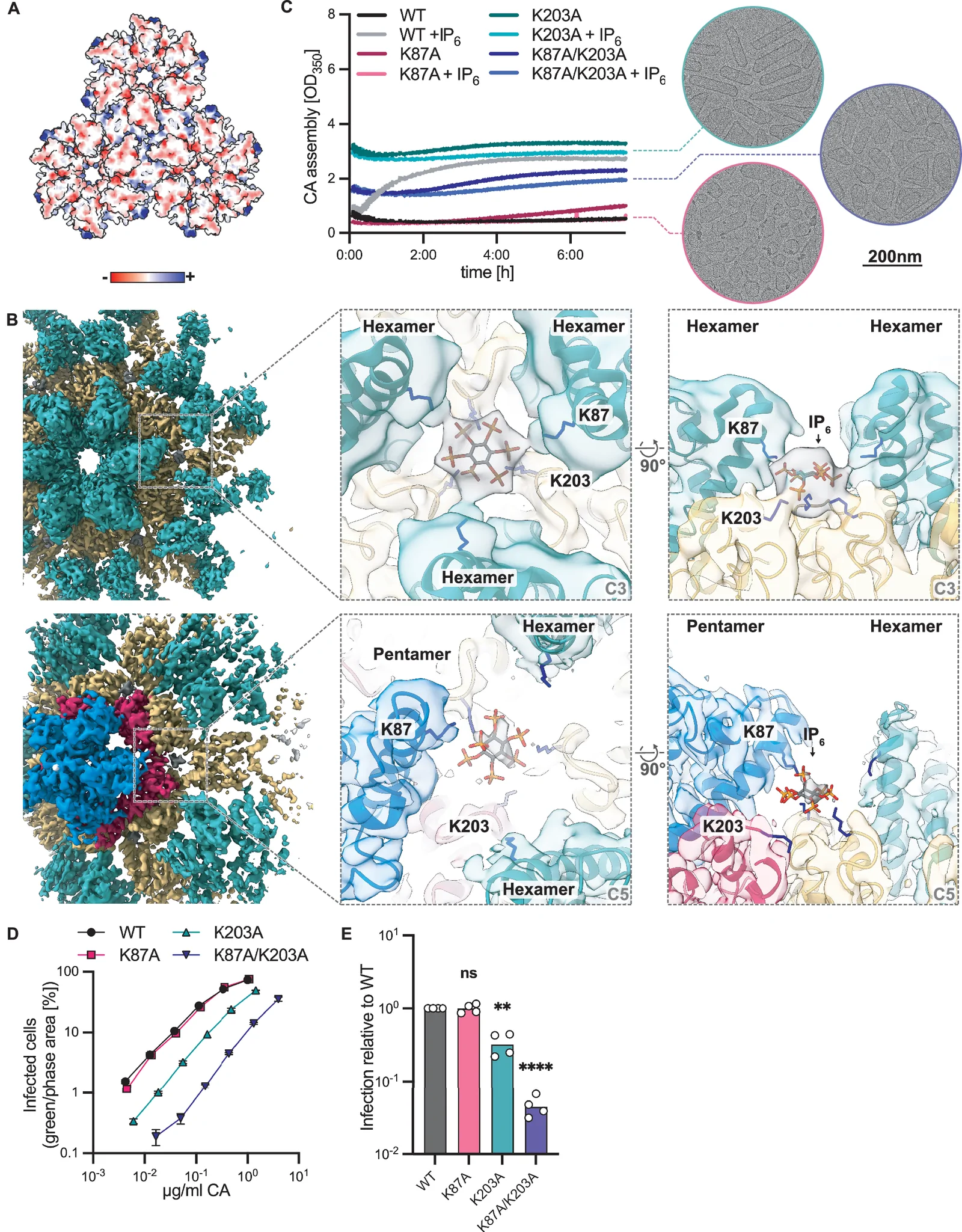
IP_6_ is bound at the threefold axis of the MPMV capsid lattice. **A** The surface electrostatic potential of a partial MPMV capsid lattice was calculated based on the MPMV hexamer structure. The surface is coloured according to the Coulomb potential, from negative (red) to positive (blue), highlighting the distinct positive pockets (blue) located at the 3-fold axis. **B** Pore-centred reconstructions of hexameric (top) and pentameric (bottom) capsomers are shown as surface representations. Magnified views show the inter-capsomer contacts of hexamers (C3 reconstruction centred at the 3-fold axis) and where a pentamer interacts with two neighbouring hexamers (C5 reconstruction centred at the capsomer pore). The density map and atomic model are colour-coded by domains for hexamers (CA_NTD_: teal, CA_CTD_: yellow) and pentamers (CA_NTD_: blue, CA_CTD_: red). The density in the 3-fold axis centre is consistent with binding a single IP_6_ molecule (grey). Adjacently located are two lysine residues, 87 and 203, which are correctly positioned to allow charged interactions with the IP_6_ phosphate groups. **C** Assembly reaction kinetics of indicated MPMV capsid mutants (500 µM) in the absence or presence of IP_6_ (1 mM) were observed by OD_350_ measurement. The shown reaction is representative of three independent replicates. Representative micrographs depict assemblies of MPMV capsid mutants. **D** Representative infections with indicated MPMV viruses are plotted as the infected percentage of the total cell area against the quantity of input virus. **E** The bar chart summarises the infectivity of mutant viruses relative to WT from four independent experiments. A one-sample two-tailed *t*-test was used to compare the mean of a mutant sample against the hypothetical mean of 1. Data are presented as mean values ± SEM. Significant differences are indicated (ns *p* > 0.05, * *p* ≤ 0.05, ** *p* ≤ 0.01, *** *p* ≤ 0.001, **** *p* ≤ 0.0001, *n* = 4 biological replicates).

Based on the polyanionic properties of IP_6_, the binding pocket is expected to be strongly positively charged. Using our MPMV hexamer reconstruction, we calculated the surface charge of a modelled capsid lattice (Fig. 3A). In contrast to the negatively charged NTD and hydrophobic C-terminal domain (CTD) environment at the HIV-1 3-fold axis^29,32^, a cluster of positive charges is evident at the vertex where three MPMV capsomers come together (3-fold axis). MPMV features two rings of positively charged residues at this position. In the MPMV capsid reconstruction, additional density can be identified at the 3-fold axis that is consistent with an IP_6_ molecule (Fig. 3B). Surrounding this putative IP_6_ density are side chain densities corresponding to residues K87 and K203 from each of the three capsomers. This suggests that IP_6_ is held at the 3-fold interface in the MPMV capsid lattice between a ring of three K203 from below the metabolite and a second ring of three K87 from above it.

We interrogated the functional involvement of these two lysine rings by mutating them to alanine and probing their capacity to support IP_6_-driven assembly in vitro (Fig. 3C). Notably, both the single and double mutations abolished the ability of IP_6_ to promote MPMV assembly. We did note, however, that in the absence of IP_6_ the mutant protein exhibits a significantly increased optical density in in vitro assembly compared to the wild-type protein. This suggests that K87 and K203 promote assembly when binding IP_6_ but disfavour assembly in the absence of IP_6_, presumably through charge repulsion. Electron micrographs of the mutant assemblies reveal morphological differences. Specifically, K87A forms smaller assemblies which approach a round shape, while K87A/K203A and especially K203A form elongated structures with large, straight lattice regions (Fig. 3C).

Consequently, these biochemical data support our structural observation that IP_6_ is coordinated at the 3-fold axis by two concentric lysine rings, K87 and K203. To substantiate the importance of this binding site for MPMV infectivity, we produced virus with K87 and K203 mutated separately or in combination (Fig. S2B). Mutant K87A retains similar infectivity to WT virus, but K203A reduces MPMV infectivity significantly (Fig. 3D, E). Combining K87A with K203A further reduces its infectivity, confirming the physiological relevance of both residues.

### MPMV is sensitive to the depletion of IP_6_ and IP_5_ in producer and target cells

**Fig. 4:**
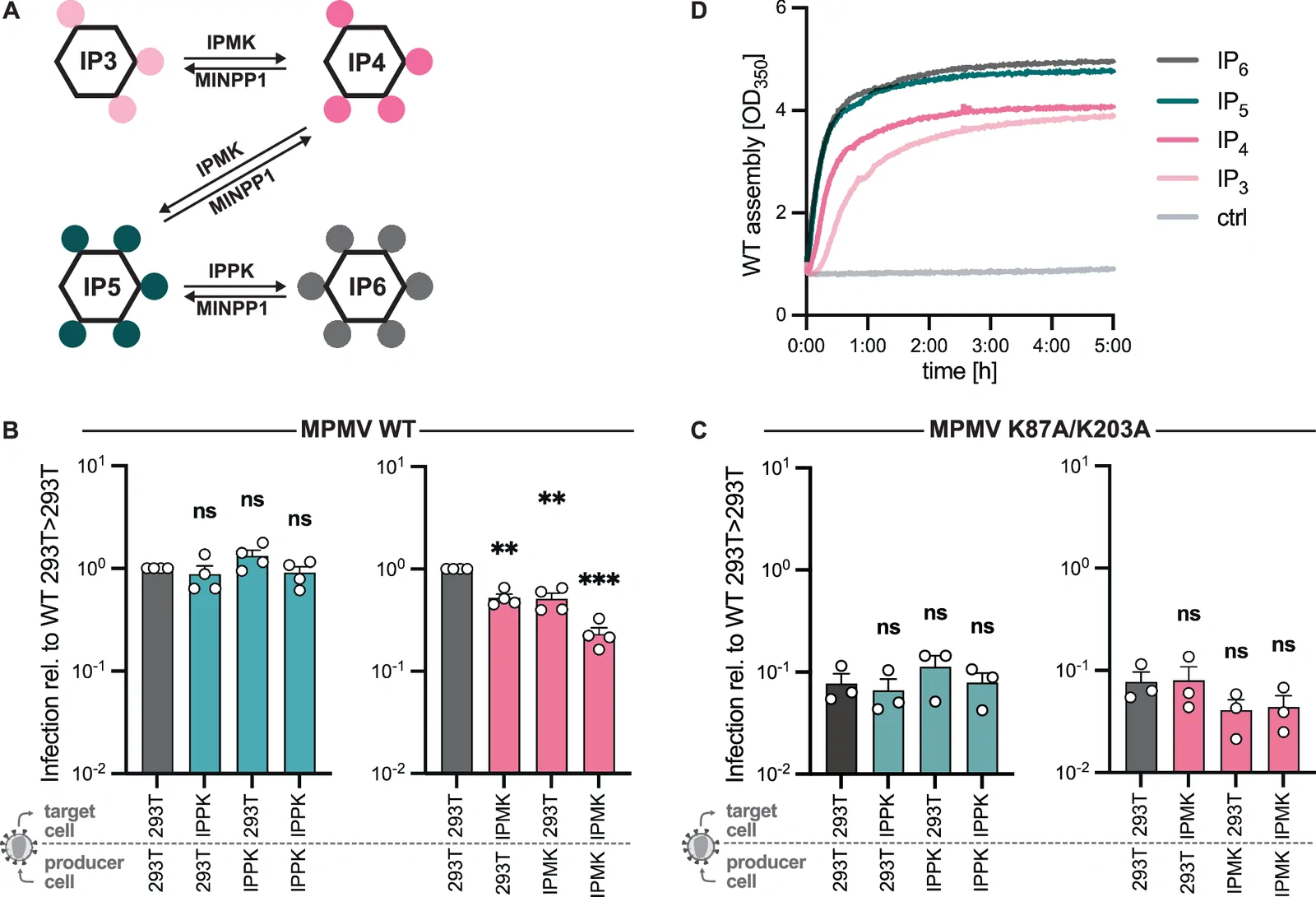
MPMV is sensitive to IP_6_ and IP_5_ depletion in producer and target cells. **A** Simplified representation of the cellular IP_6_ metabolism with key enzymes indicated. IPPK knockout (IPPK) selectively depletes cellular IP_6_; IPMK knockout in combination with MINPP1 overexpression (IPMK) depletes IP_6_ and IP_5_. WT or IP-depleted cells were infected with (**B**) WT MPMV or (**C**) K87A/K203A double mutant MPMV. Producer and target cell combinations are indicated. Infectivity is normalised to the WT control (produced in and infecting 293T parental cells), showing reduced infectivity for production in and infection of IP_5_&IP_6_-depleted IPMK cells. For viruses with a K87A/K203A double mutation, no additional decrease in infectivity was observed by depleting IPs. Data are presented as mean values +/- SEM. A one-sample two-tailed t-test was used to compare the sample mean against (**B**) the hypothetical mean of 1 or (**C**) the mutant sample mean. Significant differences are indicated (ns p > 0.05, * *p* ≤ 0.05, ** *p* ≤ 0.01, *** *p* ≤ 0.001, **** *p* ≤ 0.0001, biological replicates: WT *n* = 4, K87A/K203A *n* = 3). **D** Assembly reaction of MPMV WT capsid protein (450 µM) with the indicated inositol phosphates (150 µM). The kinetics were monitored by OD_350_ measurement and demonstrated that assembly was most effectively induced by IP_6_ and IP_5,_ and with slightly lower efficacy by IP_4_ and IP_3_. The shown reaction is representative of three independent replicates.

Previous studies have shown that reducing cellular IP_5_ and IP_6_ levels reduces HIV-1 virus production and virion infectivity^12,18–20^. Given that MPMV has an IP_6_ binding site and can use IP_6_ to build capsids, we tested whether it is similarly dependent on cellular availability of inositol phosphates (IP). We used inositol pentakisphosphate kinase knockout (IPPK KO) cells, which have reduced IP_6_ levels but retain IP_5_, and inositol polyphosphate multikinase knockout (IPMK KO) cells overexpressing the phosphatase MINPP1, which have reduced IP_5_ and IP_6_ levels (Fig. 4A)^12,19^. MPMV virion production was unaffected by IP_6_ depletion and only modestly reduced in cells depleted of IP_5_ and IP_6_ (Fig. S2C, D). This suggests that, unlike HIV-1, MPMV does not require IP_6_ for immature lattice assembly, which is consistent with previous findings that small polyanions, including IP_6_, do not facilitate immature MPMV lattice assembly^33^. Production of the double mutant K87A/K203A was similarly independent of IP-depletion, as expected, given these residues form an IP_6_ binding site only in the mature capsid (Fig. S2E, F).

Next, we tested the effect of IP-depletion on MPMV virion infectivity. Again, we used either IPPK KO cells or IPMK KO cells overexpressing MINPP1, but this time as producer cells, target cells or both. Data obtained using IPPK KO cells show that IP_6_-depletion from producer or target cells or both does not reduce MPMV infectivity. In contrast, there is a significant reduction in MPMV infectivity when viruses are either produced in or infecting IP_5_&IP_6_-depleted cells (Fig. 4B). Moreover, there is an additive effect of depleting IP_5_ and IP_6_ in both producer and target cells. The fact that there is an infectivity phenotype in both producer and target cells suggests that IP_5_ or IP_6_ can be used to assemble the capsid inside budded virions and to maintain its stability once deposited in the target cell. To test whether this IP-dependence is mediated by the IP_6_ binding site provided by K87 and K203, we repeated infectivity experiments using K87A/K203A (Fig. 4C). Strikingly, the IP-dependence of MPMV infectivity was lost in the double mutant, with no additional reduction in the infectivity of viruses either produced in or infecting IP-depleted cells, indicating that the residues are important for infectivity because they bind inositol phosphates (Fig. 4C).

While IP_5_&IP_6_-depletion in producer cells significantly reduces MPMV infectivity, we were surprised by the relatively modest magnitude of the effect. Based on the presented infection data, we believe that MPMV can substitute IP_6_ by using IP_5_ instead and may even be permissive to use further inositol phosphates. To reach a more comprehensive understanding of MPMV's IP specificity, we repeated our in vitro assembly experiments but replaced IP_6_ with a range of IPs. Remarkably, we observed that all IPs, including IP_3_, could induce rapid MPMV CLP assembly (Fig. 4D). This contrasts with HIV-1, where efficient assembly is induced exclusively by IP_5_ and IP_6_, while IP_3_ shows no activity^12^. Thus, MPMV may be less sensitive to IP_5_& IP_6_-depletion than HIV-1 because it can utilise IP_3_ or IP_4_ to build or stabilise its capsid. Taken together, the above data support a model in which MPMV uses IPs to build and stabilise its capsid, but via a completely different IP binding site from HIV-1 that is located at the 3-fold axis between capsomers.

### MLV cores bind IP_6_ at the 3-fold axis

**Fig. 5:**
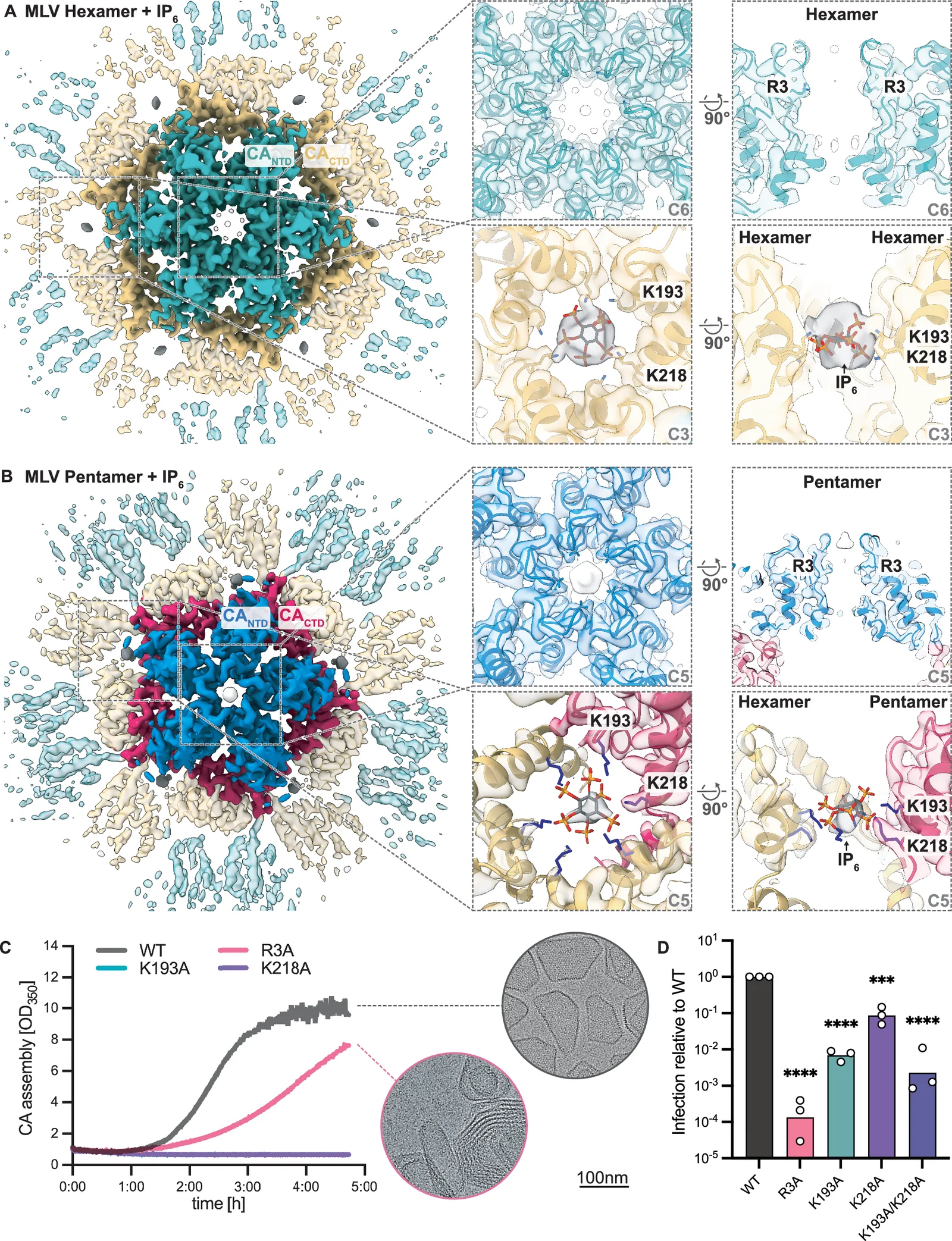
IP_6_ binds the MLV 3-fold axis and drives MLV core assembly. Single-particle reconstruction of (**A**) hexameric and (**B**) pentameric MLV capsomers from in vitro assembled MLV CLPs in the presence of 2.5 mM IP_6_. Density maps are shown as surface representations coloured by hexamer (CA_NTD_: teal, CA_CTD_: yellow) and pentamer (CA_NTD_: blue, CA_CTD_: red) with corresponding atomic models in equivalent colours. The inter-hexamer 3-fold axis (top insets) was reconstructed by recentring and C3-symmetrisation. Insets highlight the presence of an additional density at the 3-fold axes (grey) and its absence at the capsomer pore. An IP_6_ molecule was placed into the central density, visualising its coordination by Lys193 and Lys218. **C** Assembly reaction kinetics of MLV capsid protein (500 µM) in the presence of 1 mM IP_6_ were observed by OD_350_ measurement. R3A mutation delays assembly, and mutation of 3-fold axis residues completely abolishes assembly. The shown reaction is representative of three independent replicates. The shown reaction is representative of three independent replicates. Representative micrographs are shown for MLV WT and MLV R3A capsid assembled in 2.5 mM IP_6_. **D** Equivalent mutations were introduced to MLV, and their infectivity plotted relative to that of WT MLV. Data are presented as mean values ± SEM. A one-sample two-tailed *t*-test was used to compare the mutant sample mean against the hypothetical mean of 1. Significant differences are indicated (ns *p* > 0.05, * *p* ≤ 0.05, ** *p* ≤ 0.01, *** *p* ≤ 0.001, **** *p* ≤ 0.0001, *n* = 3 biological replicates).

The discovery of an alternative inositol phosphate binding site in MPMV led us to ask whether other retroviruses bind IP_6_ at the 3-fold axis. We decided to focus on MLV because it lacks a positively charged residue in helix 1 (Fig. S1A). Additionally, the architecture of the MLV 3-fold axis differs from that of HIV-1 and MPMV, forming an additional pore, though, like MPMV, it is positively charged^24^. We assembled recombinant Moloney MLV capsid protein in vitro in the presence of 2.5 mM IP_6_. Using a single-particle cryoEM pipeline, we reconstructed MLV hexamers (3.5 Å) and pentamers (3.9 Å) from CLPs. It has been speculated that IP_6_ may bind to R3 in the central capsomer pore^26^. However, we observed only weak density in the capsomer pore, next to G6 and out of reach of R3 sidechains. In contrast, stronger density is present at the 3-fold axis between MLV capsomers, analogous to that observed in the MPMV core (Fig. 5A, B), and consistent with the binding of a single IP_6_ molecule. This density was less prominent in previous studies of virions, likely because virions do not package sufficient IP_6_ to occupy all binding sites^24^. In MLV, the IP_6_ molecule is encircled and coordinated by a planar ring of six lysines, K193 and K218, supplied symmetrically by the CTDs of the three neighbouring capsomers. This is structurally distinct from the 3-fold IP_6_ binding site of MPMV where the two lysines are contributed by the NTD and CTD, are on different planes and therefore form two distinct rings.

To test whether K193 and K218 perform a similar role in MLV as K87 and K203 in MPMV, we compared in vitro CLP assembly of wild-type MLV with mutants K193A and K218A. Mutating either residue abrogated the in vitro assembly of MLV cores, confirming their importance in IP_6_ coordination (Fig. 5C). Meanwhile, the R3A mutation reduced assembly kinetics and shifted core morphologies towards multilayered assemblies. Retaining the capacity for IP_6_-driven core assembly demonstrates, however, that the R3 residue is not essential for IP_6_-driven capsid formation. Taken together, our data indicate that IP_6_ is bound by the MLV capsid and is sufficient to assemble mature cores in vitro. Structural as well as biochemical data place the IP_6_ binding site at the MLV 3-fold axis, not at R3 in the capsomer pore.

To assess whether the 3-fold axis IP_6_ binding site is important for MLV infectivity, we mutated K193 and K218 individually and together (Fig. S3A). Each mutant significantly reduced MLV infectivity, and a combined effect was observed when both K193A and K218A were mutated, reminiscent of that observed with the MPMV double mutant K87A/K203A (compare Fig. 3E and Fig. 5D). Mutants involving K193A also had a production defect and reduced processing compared to WT, likely contributing to their infectivity defect. The K218A mutation had a clear infectivity defect with minimal effects on production and processing. We hence conclude that IP_6_ binding is important for MLV infectivity and used the K218A mutant for further experiments. Consistent with the discussed literature, the mutation of R3 was fatal for virus infectivity^26^, despite not being the coordinating residue for IP_6_. Hence, R3 may have an alternative function to IP_6_ binding.

### MLV depends on IP_6_ to stabilise its capsid in the target cell

**Fig. 6:**
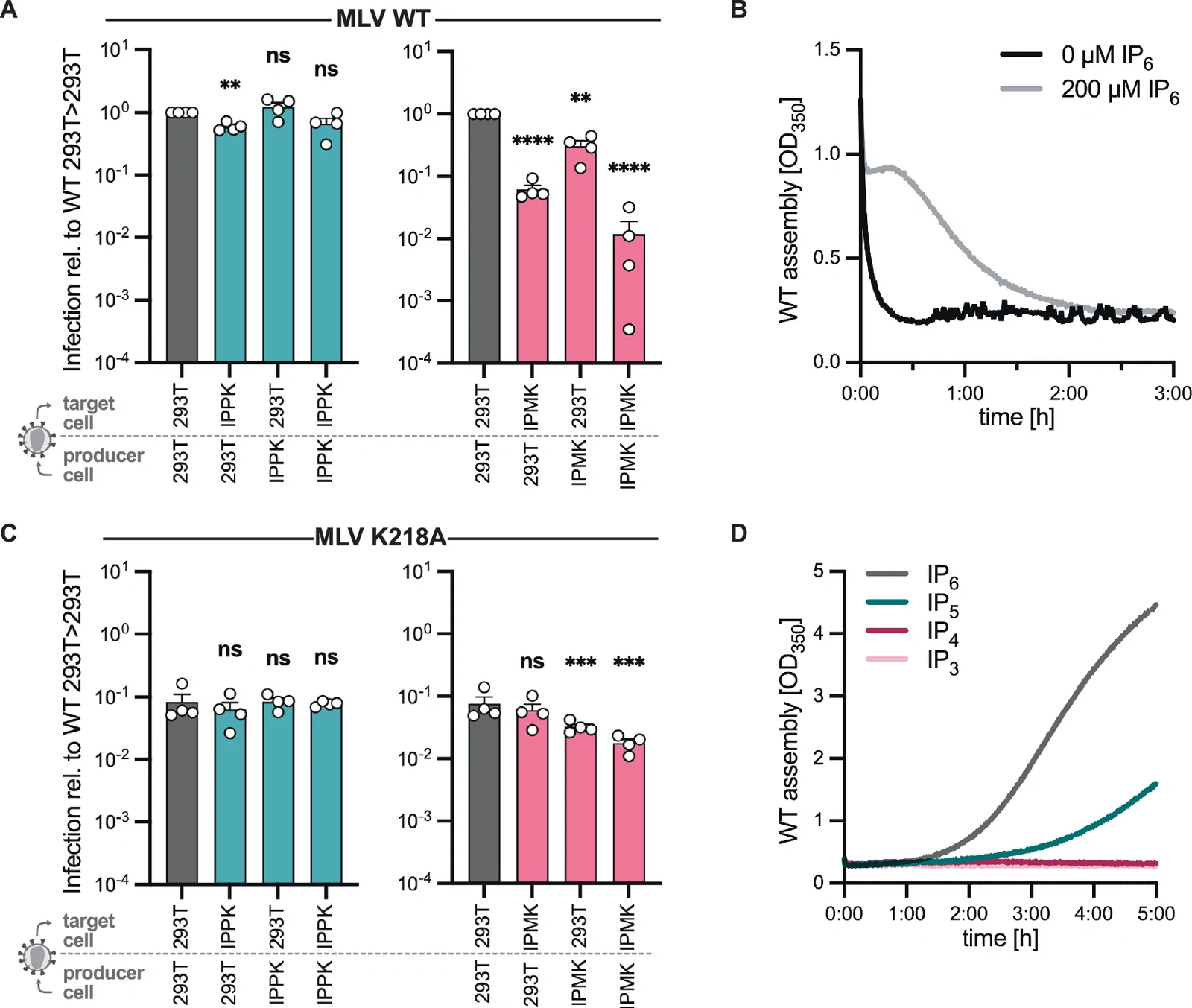
MLV is dependent on IP_6_ and IP_5_ for producing and especially stabilising its capsid in the target cell. **A** MLV WT infectivity was assayed for virus produced in and infecting IP-manipulated cells. Producer and target cell combinations are indicated (293 T: parental WT cell line, IPPK: IPPK KO resulting in specific IP_6_-depletion, IPMK: IPMK KO and MINPP1 overexpression resulting in IP_5_& IP_6_-depletion). Infectivity is normalised to the WT control (produced in and infecting 293 T parental cells), showing reduced infectivity for production in and especially for infection of IP_5_&IP_6_-depleted IPMK cells. **B** To probe IP_6_-dependence of MLV core stability, assembled MLV cores (600 µM CA, 200 µM IP_6_) were diluted 20-fold into buffers with indicated IP_6_ concentrations at t = 0 h. MLV core disassembly was monitored by OD_350,_ showing extended stability in the presence of IP_6_. The shown reaction is representative of three independent replicates. **C** IP-depletion experiments with K218A MLV show a strongly reduced IP-depletion phenotype on top of this IP_6_ binding site mutant. Data are presented as mean values ± SEM. A one-sample two-tailed *t*-test was used to compare the sample mean against (**A**) the hypothetical mean of 1 or (**C**) the mutant sample mean. Significant differences are indicated (ns *p* > 0.05, * *p* ≤ 0.05, ** *p* ≤ 0.01, *** *p* ≤ 0.001, **** *p* ≤ 0.0001, *n* = 4 biological replicates). **D** MLV WT capsid protein (500 µM) was assembled with 500 µM of the indicated inositol phosphates. Assembly kinetics were monitored by OD_350_ measurements, showing that IP_6_ induces effective assembly, IP_5_ induces less efficient assembly, whereas IP_4_ and IP_3_ do not drive assembly. The shown reaction is representative of three independent replicates.

To further characterise the IP-dependence of MLV we carried out a series of production and infection experiments in cells depleted of either IP_6_ or IP_5_&IP_6_ as described above for MPMV (Fig. 4A). Consistent with published data, we did not observe any reduction in viral production of WT MLV in IPPK KO cells (Fig. S3B, C). Fewer virions were produced in IPMK KO cells overexpressing MINPP1 and this coincided with reduced overall CA expression in the cell lysate (Fig. S3B, C). MLV infectivity was largely unaffected whether the virus was produced in IPPK KOs, infecting IPPK KOs, or both (Fig. 6A). In contrast, when IPMK KOs overexpressing MINPP1 were used as the producer cells, target cells or both, there was a significant decrease in WT MLV infectivity (Fig. 6A). This contrasts with published data where MLV produced in IPMK KO cells had no significant infectivity defect^26^. We observed not only a defect in IP_5_&IP_6_-depleted producer cells but also that this is potentiated when the produced viruses are used to infect IP_5_&IP_6_-depleted target cells (Fig. 6A). This synergistic effect is likely because capsids that manage to form in IP_5_&IP_6_-depleted virions will be even more sensitive to reduced IP_5_&IP_6_ levels in target cells, falling apart before replacement molecules can be scavenged. Of note, the effect of IP_5_&IP_6_-depletion in target cells was more pronounced than in producer cells, suggesting an especially high dependence of MLV on inositol phosphates to stabilise the capsid once released into the target cell.

To test the importance of IP_6_ for maintaining MLV capsid stability, we diluted preassembled CLPs into a buffer that either has IP_6_ or lacks it, thereby testing the effect of rapidly lowering the IP_6_ concentration on CLP stability and mimicking core release into the target cell (Fig. 6B). The presence of IP_6_ in the dilution buffer extended the half-life of cores from 2.5 min to around 45 min. This result is consistent with the marked loss of infectivity observed when WT MLV infects IPMK KO cells overexpressing MINPP1. Together, this suggests that IP_6_ is crucial to prevent the collapse of MLV cores when they are released into the target cell upon viral fusion.

To provide further evidence linking IP binding by the lysines at the 3-fold axis with MLV’s IP_5_&IP_6_-dependence, we compared the sensitivity of WT and K218A viruses to IP-depletion (Fig. 6A, C). This allowed us to determine whether the loss of infectivity upon mutation is specifically due to disrupted IP_5_ and IP_6_ interaction. K218A was chosen because it is more infectious than K193A and therefore there is more potential to detect an additive effect in IP-depleted cells. K218A infectivity was not reduced in IPMK KO cells overexpressing MINPP1, in contrast to WT virus where there is a > 1-log drop in infectivity. Similarly, K218A infectivity was less affected than WT when IPMK KOs overexpressing MINPP1 were used as both producer and target cells. The presence of some residual effect is expected, as K193 remains able to bind IP_6_, and this is consistent with the reduction in infectivity observed when K193A is added to K218A. Together, these results show that MLV’s functional use of IP_6_ is mediated by the 3-fold axis binding site.

Taken together, the cellular IP-depletion data suggest that IP_5_ and IP_6_ can be used by MLV to both build its capsid and to stabilise it once deposited in target cells. This result is broadly similar to that observed for MPMV, although in MLV the phenotype of IP_5_&IP_6_-depletion is much more pronounced. To explore this difference further, we tested the ability of different IP species to assemble MLV CLPs in vitro. Both IP_5_ and IP_6_ effectively assembled MLV CLPs, albeit IP_5_ displayed slower kinetics, but no assembly was observed when using IP_3_ or IP_4_ (Fig. 6D). This contrasts with MPMV, which can use IP_3_-IP_6_. Thus, the different degree of infectivity loss observed upon cellular IP_5_&IP_6_-depletion when comparing MLV to MPMV may be because MLV is more restricted in the IP species it can use.

## Discussion

Inositol hexakisphosphate (IP_6_) is an essential cofactor in HIV-1 replication, where it assembles and stabilises the capsid by binding two charged rings within the central pore of capsomers^3,11^. While this mechanism is well-characterised in lentiviruses, data from other retroviral genera remain limited^34^.

Here, we describe data demonstrating the role of IP_6_ as a stabilising factor of the beta-retrovirus MPMV and the gamma-retrovirus MLV. This suggests that IP_6_ usage may be more broadly conserved across retroviruses than previously thought. However, unexpectedly, our data reveal a previously unknown IP_6_ binding site at the 3-fold axis of the retroviral capsid lattice. This finding contrasts with a recent study suggesting that MLV residue R3 coordinates IP_6_ for capsid assembly in the capsomer pore^26^. However, our results show that R3A can assemble in the presence of IP_6_ and that while R3A is non-infectious, MLV virions retain some infectivity when produced in, or added to, IP_6_-depleted cells. The severity of the R3A infection phenotype therefore suggests that it has a critical function distinct from IP_6_ binding. This may be the binding of a different cofactor or the import of dNTPs for reverse transcription, analogous to the HIV-1 pore^4^.

Combining our single-particle EM structures of MLV and MPMV with biochemical experiments, we have defined the molecular interactions that coordinate IP_6_ at this new interface. While both viruses bind IP_6_ at the 3-fold axis, they utilise different binding mechanisms. In MPMV, there are two rings of three lysines, one from the NTD and one from the CTD below, with IP_6_ coordinated in between. In MLV, two lysines from the CTD form a single planar ring of six lysines with IP_6_ in the centre.

These different binding mechanisms may explain why the two viruses differ in their dependence on specific IP species. MPMV can use any IP from IP_3_-IP_6_ to drive in vitro capsid assembly whilst MLV needs either IP_5_ or IP_6_. Likewise, MPMV is less affected by IP_5&_ IP_6_-depletion in either producer or target cells than MLV. We hypothesise that these differences are because MPMV can use its two separate lysine rings to coordinate two IP_3_ or IP_4_ molecules in place of a single IP_5_ or IP_6_, whereas MLV, with its single ring, can only bind a single IP molecule (Fig. S1C).

A key question raised by our work is why MPMV and MLV use IP_6_ to stabilise an inter-capsomer interface whilst HIV-1 uses IP_6_ to stabilise the capsomers themselves. Linking these structural variations with infectivity data reveals several key differences between the viruses. MLV and MPMV require IPs in both producer and target cells, but with a stronger dependence on IP_6_ in target cells. In contrast, HIV-1 is insensitive to IP depletion in target cells unless combined with capsid-destabilising mutations^18,19,21^. HIV-1 is instead critically dependent upon IP levels in producer cells^19^, with the immature HIV-1 lattice packaging these molecules during assembly to ensure a sufficient concentration inside virions to facilitate capsid maturation^12^. There is no evidence that either MLV or MPMV package IP_6_, and published data report low levels of IP_6_ in MLV virions^26^. The absence of IP_6_ packaging may explain why the density for the metabolite is weak at the 3-fold axis in capsid structures solved from virions^24^, while MLV’s sensitivity to IP6 levels in target cells suggests binding to the capsid also takes place post-entry. The different IP-dependencies in producer versus target cells may also be influenced by the intra-capsomer vs inter-capsomer interfaces binding IP_6_ with different affinities. Stronger IP_6_ binding by HIV-1 within capsomers would make it more dependent upon the metabolite during assembly but less dependent on replacing it post-assembly in target cells. Conversely, weaker IP_6_ binding in MPMV and MLV correlates with their reduced dependence on the metabolite during assembly but increased sensitivity to its levels in target cells.

Our results highlight that IP_6_ may be a key cofactor utilised by many retroviruses. Two genera of orthoretrovirinae remain to be investigated but AlphaFold3-based prediction suggests that delta-retroviruses like HTLV-1 have residues at both the central capsomer pore and the 3-fold axis that could bind IP_6_, while the epsilon-retrovirus WDSV features a charged ring that could bind IP_6_ in the capsomer pore. Conversely, spumaviruses do not require IP_6_ for assembly^35^ and the common orthoretroviral ancestor Ty3 lacks an obvious IP_6_ binding site^36,37^. This suggests that IP_6_ usage may have evolved convergently in multiple viral species and that utilising a dissociable noncovalent ligand to regulate capsid assembly and disassembly provides retroviruses with a significant functional advantage. Why different retroviruses have evolved different IP_6_ interfaces also remains to be determined. However, we hypothesise that using the metabolite to stabilise the capsid lattice within vs between capsomers has different structural and functional consequences that may be linked to key differences in viral behaviour, such as their mechanism of nuclear genome delivery and uncoating.

## Methods

### Capsid protein purification

Recombinant MPMV and MLV (MoMLV) capsid proteins were expressed from a pOPT vector in *E. coli* C41(DE3) cells grown in TB medium. The CA-encoding sequences were amplified with additional overhangs from virus production plasmids and cloned into pOPT vectors using Gibson assembly^38,39^. Expression was induced at mid-log phase with 1 mM IPTG, followed by overnight incubation at 18 °C. Cells were harvested by centrifugation (4500 × *g*, 30 min), resuspended in lysis buffer (50 mM Tris-HCl pH = 8.0, 200 mM NaCl, 1 mM DTT, cOmplete protease inhibitor (Roche), 20% BugBuster (Millipore)), lysed by sonication, and clarified by centrifugation (45,000 × *g*, 40 min, 4 °C).

The soluble fraction was subjected to ammonium sulphate precipitation at 15% (MPMV) or 25% (MLV) saturation. Precipitated proteins were pelleted (20,000 × *g*, 20 min, 4 °C), resuspended in Buffer A (50 mM Tris-HCl pH=8.0, 1 mM DTT), dialysed against Buffer A, and clarified.

MLV and MPMV capsid proteins were purified by ion exchange chromatography on a 20 mL Q HP column. Proteins were eluted using a linear gradient over five column volumes into Buffer B (50 mM Tris-HCl pH=8.0, 1 M NaCl, 1 mM DTT). Capsid protein-containing fractions were pooled and further purified by size exclusion chromatography (Superdex 75) in SEC buffer (20 mM Tris-HCl pH=8.0, 20 mM NaCl, 1 mM TCEP). Fractions were analysed by SDS-PAGE, and peak fractions were pooled and concentrated.

### Capsid assembly assays

Capsid proteins were pre-diluted to yield identical concentrations across all tested mutants. Assembly reactions were initiated by the addition of IP_6_ or indicated inositol phosphates as part of a 10-fold concentrated buffer. Buffer addition adjusted the reaction conditions to 50 mM Tris-HCl pH=8.0, 2 mM TCEP and IP concentrations as specified for each experiment. Reactions were monitored by measuring optical density at λ = 350 nm (OD₃₅₀) using a PHERAstar FSX plate reader (BMG Labtech) at 30 s intervals over the course of several hours. Shaking was applied between measurements to ensure sample homogeneity.

### Capsid disassembly assay

Capsid protein was preassembled as described for the capsid assembly assay, yielding a precise saturation of IP_6_ binding sites (600 µM capsid protein, 200 µM IP_6_). The assembly reaction was then diluted 20-fold into buffer (50 mM Tris-HCl pH = 8.0, 2 mM TCEP) containing either 0 µM or 200 µM IP_6_.

### Cells and Plasmids

HEK293T cell lines were cultured in Dulbecco’s modified Eagle’s medium (DMEM) with 10% FBS, 2 mM L-glutamine, 100 U/ml penicillin, and 100 µg/ml streptomycin (GIBCO) at 37 °C with 5% CO_2_). Replication-deficient VSV-G pseudotyped virions were produced in HEK293T cells using the lentiviral packaging plasmid pMD2.G, which encodes VSV-G envelope protein (Addgene plasmid # 12259) alongside either pSARM^38^ for MPMV or pCMVi and pCNCG (for MLV)^39^. In all cases, CA mutagenesis was performed using the QuikChange method (Stratagene). Cells knocked out for IPMK or IPPK are described and characterised in refs. ^12,19^.

### Virus production and infection

Viruses were produced from 5 ×10^5^ cells per well of a 6-well plate, plated the day before. Transfection mixtures were made using 60 µl Opti-MEM (GIBCO), 300 ng pMD2.G, 4.8 µl FuGENE-6 (Promega) with either, 1.5 µg pSARM, or 600 ng pCMVi and 600 ng pCNCG. Mixtures were incubated at room temperature for 15 min and then added entirety to a well of a 6-well plate. Viral supernatants were harvested 48-72 h post-transfection, filtered through a 0.45 µm filter and stored at −80 °C. For infection, 293T cells were seeded at 10^4^ cells per well into 96-well plates and left to adhere overnight. Indicated amounts of virus were added, and the plates were scanned every 8 h for up to 72 h in an IncuCyte® (Sartorius) to identify GFP-expressing cells.

### Virus quantification

MLV virus was quantified based on the RT enzyme concentration using a qRT-PCR as described previously with slight alterations^40^. In brief, 5 µl of viral supernatant was mixed with 5 µl lysis buffer (0.25% Triton X-100, 50 mM KCl, 100 mM Tris-HCl (pH = 7.4), 40% glycerol) and 0.1 µl RNase Inhibitor and incubated for 10 min at room temperature before diluting to 100 μL with nuclease-free water. 2 µl of lysate were added to 5 µl TaqMan Fast Universal PCR Mix, 0.1 µl MS2 RNA, 0.05 µl RNase Inhibitor and 0.5 µl MS2 primer mix, to a final volume of 10 µl. The reaction was run on an ABI StepOnePlus Real Time PCR System (Life Technologies), with an additional reverse transcription step (42 °C, 20 min).

MPMV preparations were quantified based on CA concentration using automated western blotting on Jess, as detailed in the methods section below. The bands were quantified by densitometry with purified CA protein as a standard.

### Western blotting

Viral samples were separated by capillary electrophoresis using the separation module for 12-230 kDa (SM-W008) on Jess (Simple Western, Protein Simple) according to manufacturer’s instructions. Anti-MPMV CA and anti-MLV CA antibodies were generated in-house by immunisation of mice with recombinant CA protein and were used at 1:100 dilution to ensure saturation and accurate quantification. The target was detected via HRP using an anti-mouse detection module (DM-002). Results were analysed using Compass software (Protein Simple). Alternatively, for Western blotting, viral samples were separated by SDS-PAGE and transferred to nitrocellulose and probed with the antibodies described above at a 1:1000 dilution, followed by an anti-mouse HRP-linked secondary antibody, and detected with ECL reagent.

### Statistical analysis

Unless otherwise indicated, statistical analyses were Student’s t-tests and performed using GraphPad Prism 11 software (GraphPad). Error bars depict the mean ± SEM unless indicated otherwise.

### Cryo-EM grid preparation and data collection

For both MPMV CA and MLV CA, 4 µL of core-like particle preparations were applied to the carbon side of glow-discharged C-Flat CF-2/2-3 Cu 300 mesh grids. Grids were blotted from both sides and plunge-frozen in liquid ethane using a Thermo Fisher Scientific Vitrobot Mark IV (100% humidity, 4 °C, blot time 3 s, blot force 3).

For single-particle analysis, data were acquired using a Thermo Fisher Scientific Titan Krios G3 transmission electron microscope, operated at 300 keV, equipped with a Falcon 4 detector using EPU software. CryoEM movies were collected at 96,000x nominal magnification, corresponding to a calibrated pixel size of 0.84 Å, with a total accumulated dose exposure of 40 e-/Å^2^ and a defocus range of −3.2 to −1.2 µm in 0.2 µm increments.

### CryoEM data processing

Single-particle reconstruction of MPMV and MLV hexamers and pentamers broadly followed pipelines used for HIV-1 CA CLPs^29–31^. CryoEM movies were first motion-corrected and dose-weighted in RELION 5.0.0 using MotionCor2^41^, and then imported into cryoSPARC v4.4.1^42^ where CTF estimation was performed using Patch CTF. Automated particle picking was performed using crYOLO v1.9.9^43^, for which the model was trained by manual annotation of ~20 motion-corrected micrographs, with picks being distributed across the CLP surface. Picked coordinates were then imported into cryoSPARC, where particles were initially extracted with a 384-pixel box size, Fourier-cropped to 192 pixels (Fig. S4–8).

An initial round of 2D classification was performed to remove low-quality particles. For capsomer-centred reconstructions, the remaining particles were subjected to parallel rounds of heterogeneous refinement, which were either initialised with a hexameric or pentameric reference, enforcing C6 and C5 symmetry, respectively (Fig. S4, S6, S8). For both hexamers and pentamers, output classes were evaluated by the apparent map quality and nominal resolution. Selected classes were then pooled and subjected to a single round of non-uniform refinement^42^ to generate an initial reconstruction, after which any duplicate particles were removed.

To further improve the reconstruction quality and resolution, additional rounds of heterogeneous refinement, local refinement, local CTF estimation and non-uniform refinement were then performed in cryoSPARC. Particles were then exported into RELION 5.0.0 where Bayesian polishing^44^ was performed, before being re-imported to cryoSPARC for a final round of refinement. Summary statistics for the final cryo-EM reconstructions are provided in Table 1.

**Table 1:** CryoEM data collection and map resolution details

	MPMV hexamer	MPMV 3FA	MPMV pentamer	MPMV E26A hexamer	MPMV E26A pentamer	MLV hexamer	MLV 3FA	MLV pentamer
**Data collection**								
Microscope	Titan Krios G3			Titan Krios G3		Titan Krios G3		
Detector	Falcon 4			Falcon 4		Falcon 4		
Magnification	96,000×			96,000×		96,000×		
Voltage (kV)	300			300		300		
Electron exposure (e–/Å2)	40			40		40		
Defocus range (μm)	3.2–1.2			3.2–1.2		3.2–1.2		
Pixel size (Å)	0.84			0.84		0.84		
Movies (no.)	12,534			13,728		12,277		
**Data Processing**								
Initial particle images (no.)	3,279,458			1,320,315		9,098,084		
Symmetry imposed	C6	C3	C5	C6	C5	C6	C3	C5
Final particle images (no.)	538,682	160,022	47,078	63,876	20,460	86,595	150,880	54,857
Map resolution (Å)	3.19	4.1	3.26	3.76	4.36	3.51	4.2	3.89
FSC threshold	0.143	0.143	0.143	0.143	0.143	0.143	0.143	0.143

For 3-fold axis-centred reconstructions of MPMV and MLV hexameric lattice, initial references were derived by centring the hexameric reconstruction on the 3-fold axis (volume alignment tools) and performing a reconstruction with C3 symmetry applied. These were used to initialise new sets of heterogeneous refinement using the initially selected 2D classes for MPMV and pre-selected and recentred hexamer particles for MLV. Heterogeneous refinement-derived 3D classes were visually inspected and pooled based on their quality and then further refined in a similar manner to the capsomer-centred capsid lattice reconstruction (Fig. S5, S7).

### Model building and visualisation

Initial atomic models were generated using AlphaFold3^45^. Models were first rigid-body fit into corresponding cryo-EM density using ChimeraX^46^, after which the models were manually refined in Coot^47^. Multiple rounds of real space refinement were then performed in Phenix-1.21.2^48^. Final model validation statistics and the map-to-model Fourier shell correlation (FSC) were calculated in Phenix-1.21.2 and are reported in Table 2. Final models were visualised and analysed in ChimeraX.

**Table 2:** CryoEM summary table of model building and refinement

	MPMV hexamer	MPMV 3FA	MPMV pentamer	MPMV E26A hexamer	MPMV E26A pentamer	MLV hexamer	MLV 3FA	MLV pentamer
**Refinement**								
Initial model used	AF3	AF3	AF3	AF3	AF3	AF3	AF3	AF3
Model resolution (Å)	3.5	4.6	3.5	4.1	4.8	3.8	4.8	4.3
FSC threshold	0.5	0.5	0.5	0.5	0.5	0.5	0.5	0.5
Map sharpening B factor (Å^2^)	182.9	75	136.3	156.7	196.2	133	150.7	147.2
Model composition								
Non-hydrogen atoms	18888	12534	26955	10870	10735	22050	5544	16920
Protein residues	1242	816	1775	1660	1392	1392	696	2130
Ligands	0	0	0	0	0	0	0	0
B factors (Å^2^)								
Protein	69.66	156.91	65.68	92.66	176.2	67.14	166.9	82.22
Ligand	-	-	-	-	-	-	-	-
R.m.s. deviations								
Bond lengths (Å)	0.004 (0)	0.005 (0)	0.005 (0)	0.004 (0)	0.004 (0)	0.004 (0)	0.004 (0)	0.004 (0)
Bond angles (°)	0.895 (0)	0.920 (0)	0.903 (0)	0.894 (0)	0.941 (0)	0.870 (0)	0.959 (0)	0.973 (0)
Validation								
MolProbity score	1.57	1.85	1.37	1.72	1.14	1.36	1.13	1.57
Clashscore	0.95	7.51	0.74	3.87	3.46	2.22	3.4	2.43
Rotamer outliers (%)	3.05	0	2.16	2.41	0	1	0	2.98
Ramachandran plot								
Outliers (%)	0	0	0.29	0	0	0	0	0.24
Allowed (%)	6.4	6.87	5.22	3.74	1.92	5.22	1.74	2.86
Favored (%)	93.6	93.13	94.49	96.26	98.08	94.78	98.26	96.9
*Q*-Score	0.51	0.33	0.49	0.43	0.32	0.47	0.34	0.41

### Reporting summary

Further information on research design is available in the Nature Portfolio Reporting Summary linked to this article.

## Supplementary information

Supplementary Figs.

Reporting Summary

Transparent Peer Review file

## Source data

Source Data

## Data Availability

The cryo-electron microscopy density maps have been deposited in the Electron Microscopy Data Bank (EMDB) under accession codes EMD-56387 (MoMLV 3FA), EMD-56388 (MoMLV Hex), EMD-56389 (MoMLV Pent), EMD-56390 (MPMV 3FA), EMD-56394 (MPMV Hex), EMD-56393 (MPMV Pent), EMD-56392 (MPMV E26A Hex), EMD-56391 (MPMV E26A Pent). The corresponding molecular models have been deposited in the Protein Data Bank (PDB) under accession codes 9TX4 (MoMLV 3FA), 9TX5 (MoMLV Hex), 9TX6 (MoMLV Pent), 9TX7 (MPMV 3FA), 9TXB (MPMV Hex), 9TXA (MPMV Pent), 9TX9 (MPMV E26A Hex), 9TX8 (MPMV E26A Pent). Source data are provided with this paper.

## Code availability

No custom code was generated for this study. All data were processed using publicly available software packages, as described in the Methods.
