# Supplementary material for: Retroviruses use different IP6 binding mechanisms to alter the properties of their capsids
Source: Nat Commun. 2026 Aug 21;17:10005. doi: 10.1038/s41467-026-76510-7 (PMC13590620; doi:10.1038/s41467-026-76510-7)
Supplement: Supplementary file 1 [file 41467_2026_76510_MOESM1_ESM.pdf]

Retroviruses use different IP_6 binding mechanisms to alter the properties of their capsids

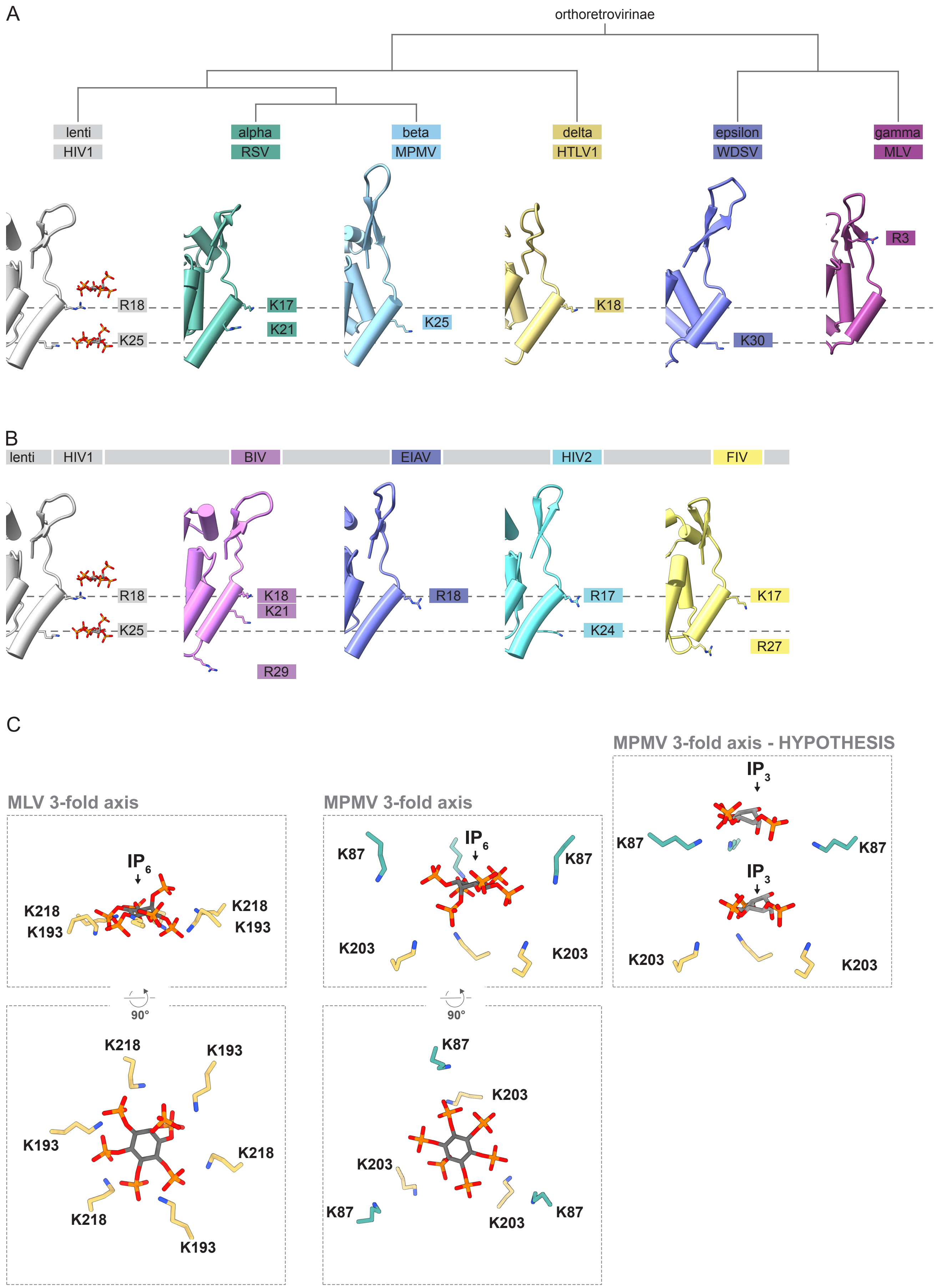

Supplementary Figure 1. Comparison of pore residues across retroviruses and IP binding sites between MPMV and MLV. (A) Phylogenetic tree adapted from Mayer et al., 2025 [49] indicates the points of genetic divergence of genera in the orthoretrovirinae family. For each genus, a member's capsid was predicted with AlphaFold3 and vertically aligned.

(B) AlphaFold3 predictions of lentiviral capsids are depicted and vertically aligned. Positively charged amino acid residues in helix 1, facing the central capsomer pore, are shown and labelled. Dashed lines indicate the relative positions of HIV-1 R18 and K25. (C) The IP_6 binding sites in the 3-fold axis of the MLV and MPMV capsid lattices are depicted, with only the IP_6-coordinating side chains shown. For MPMV, a hypothetical model depicts a proposed binding mode in which each lysine ring binds one IP_3 molecule instead of coordinating one IP_6 molecule together.

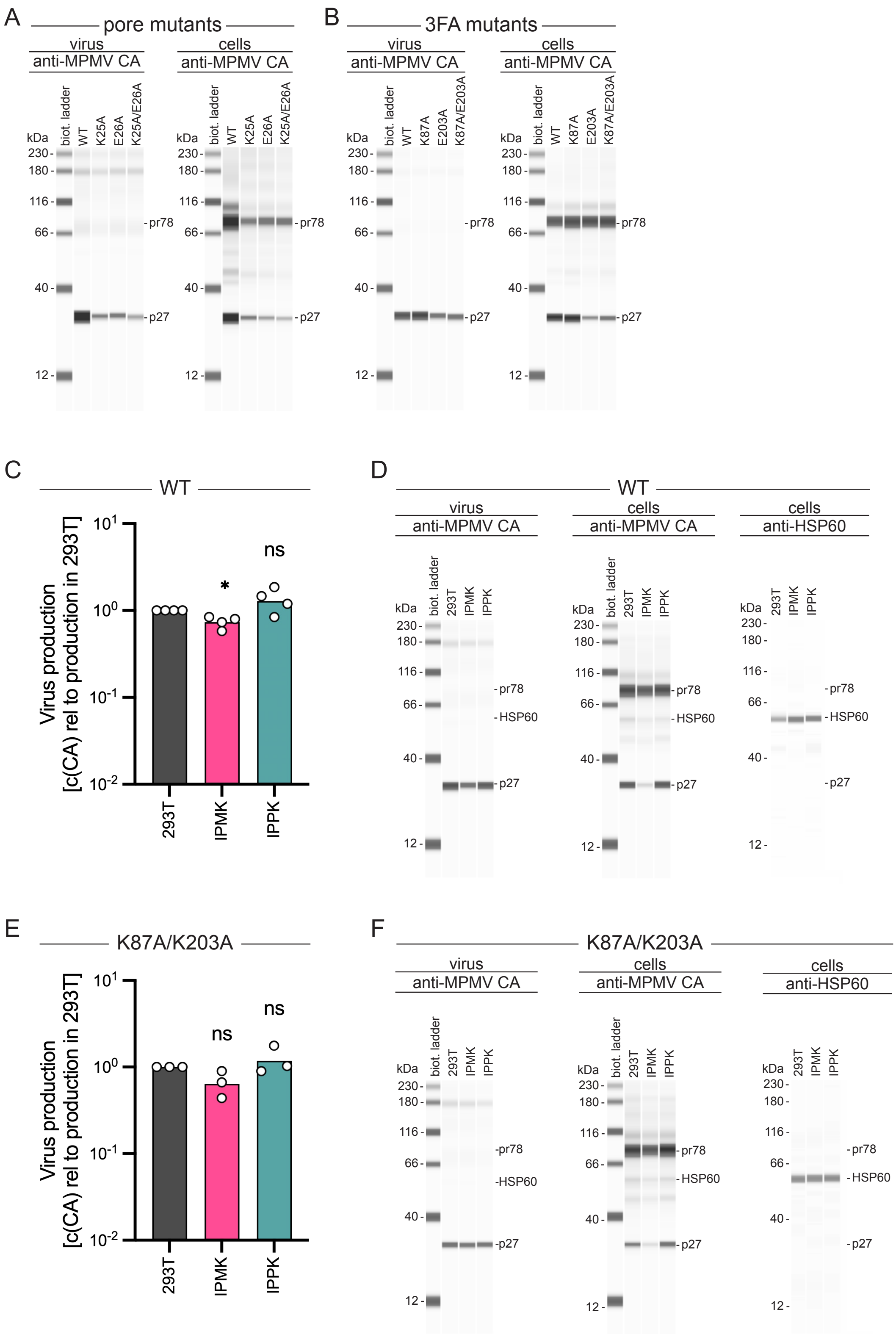

Supplementary Figure 2. MPMV production for tested mutations or production in IP-depleted cells. MPMV CA (p27) concentrations were determined by automated Western Blotting on Jess. Representative Western Blots with anti-MPMV CA polyclonal antibodies in virus-containing supernatant and whole producer cell extract are shown. Data is presented for wild-type, (A) pore mutants, and (B) 3-fold axis mutants showing reduced expression but equivalent virus release and processing of mutant MPMV compared to WT MPMV. MPMV was produced in parental cell line (293T) or IPMK knockout cells with MINPP1 overexpression (IPMK) or IPPK knockout cells (IPPK). (C) The released capsid quantities differ only minimally from wild-type MPMV. A one-sample two-tailed t-test was used to compare the mean of the sample against the hypothetical mean of 1. Significant differences are indicated (ns $p > 0.05$, * $p \leq 0.05$, ** $p \leq 0.01$, *** $p \leq 0.001$, **** $p \leq 0.0001$, n = 4 biological replicates). (D) Viral supernatant and whole cell extract were analysed by Western Blotting with anti-MPMV CA polyclonal antibodies, demonstrating consistent release of MPMV in IP-modified cells.

(E,F) Analogous data are shown for the K87A/K203 mutant. A one-sample t-test was used to compare the mean of the sample against the hypothetical mean of 1. Significant differences are indicated (ns $p > 0.05$, * $p \leq 0.05$, ** $p \leq 0.01$, *** $p \leq 0.001$, **** $p \leq 0.0001$, n = 3 biological replicates). For wild-type and mutant MPMV blots, anti-HSP60 blotting was included as a housekeeping control.

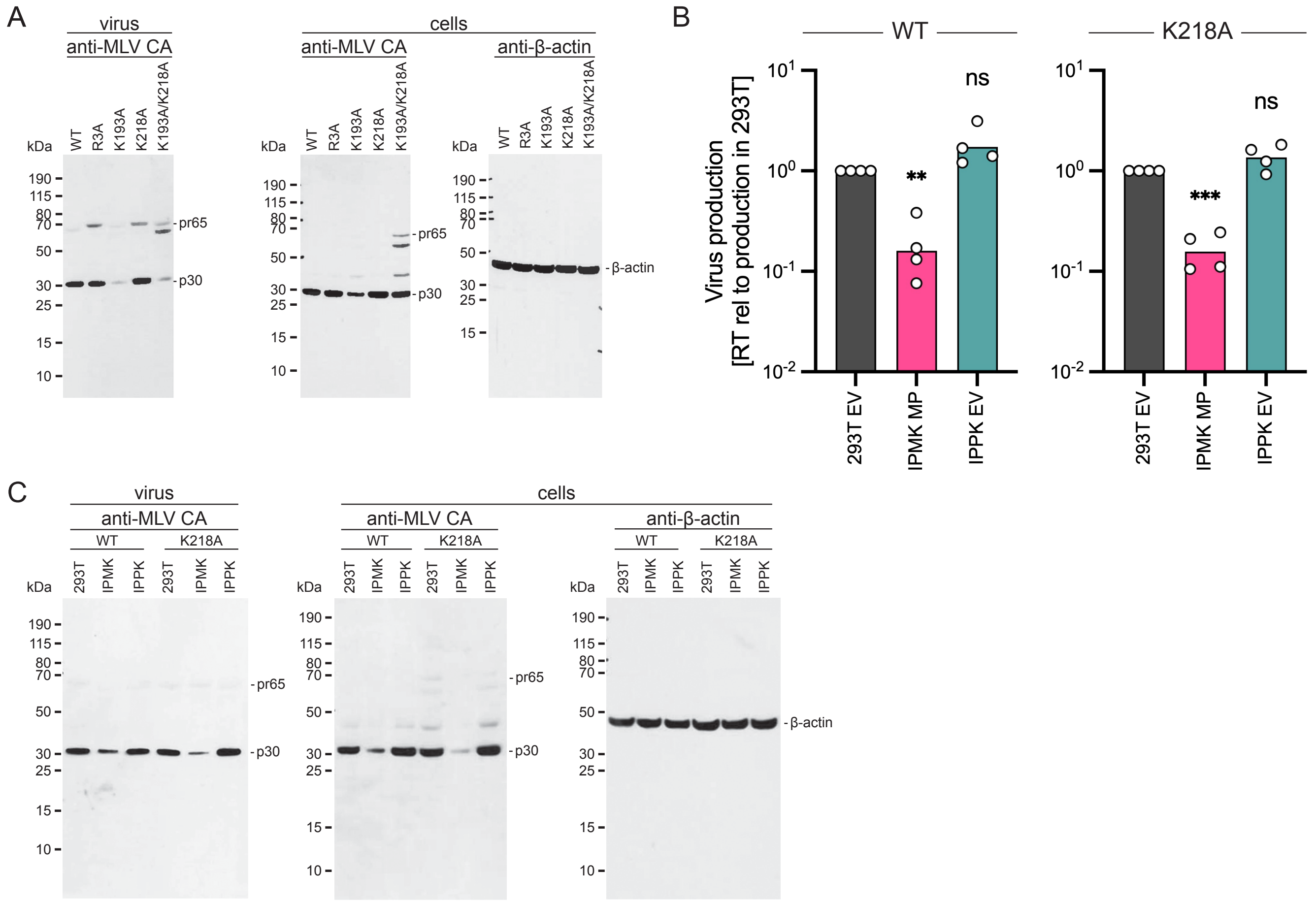

Supplementary Figure 3. MLV production for tested mutations or production in IP-depleted cells. (A) Production of wild-type and mutant MLV was assessed by Western blotting. MLV CA was detected in whole-cell extracts and corresponding virus-containing supernatants using a polyclonal anti-MLV CA antibody. The mutants K193A, and K193A/K218A have reduced expression levels and incomplete processing. The production of R3A and K218A mutants is comparable to that of WT MLV. β-actin served as a loading control. (B,C) MLV was produced in parental cell line (293T) or IPMK knockout cells with MINPP1 overexpression (IPMK) or IPPK knockout cells (IPPK). (B) Production of wild-type and K218A MLV generated in the indicated cell lines was quantified by in vitro reverse transcriptase (RT) activity and normalised to wild-type MLV produced in parental 293T cells. A one-sample two-tailed t-test was used to compare the mean of a sample against the hypothetical mean of 1. Significant differences are indicated (ns $p > 0.05$, * $p \leq 0.05$, ** $p \leq 0.01$, *** $p \leq 0.001$, **** $p \leq 0.0001$, n= 4 biological replicates). (C) The corresponding Gag expression, release, and processing was assessed by Western blotting against MLV CA (p30). IPMK KO cells overexpressing MINPP1 showed reduced Gag expression levels, consistent with reduced production measured by RT quantification, and this was normalised for infection experiments. No release or processing defect was apparent. Production in IPPK cells was comparable to that of parental cell lines. β -actin was included as a loading control.

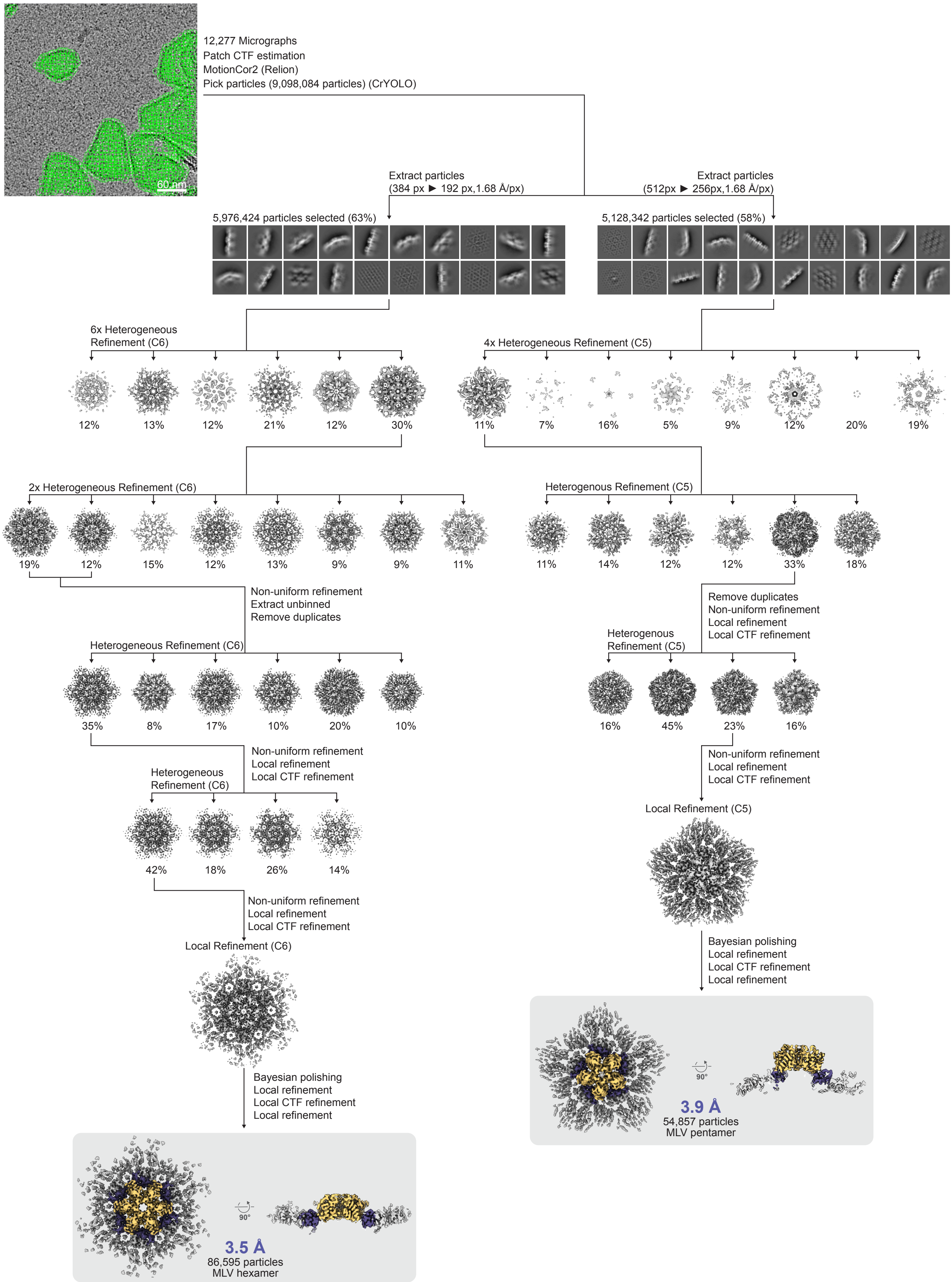

Supplementary Figure 4. Cryo-EM data processing workflow for MLV hexamer and pentamer structures from CLPs. Particle picking across the CLP surface was automated in crYOLO. High-quality particle picks were selected in 2D classification, and hexamer and pentamer positions were sorted by heterogeneous refinement. In the final reconstruction, the CA_{NTD} and CA_{CTD} of central capsomers are shown in yellow and purple, respectively.

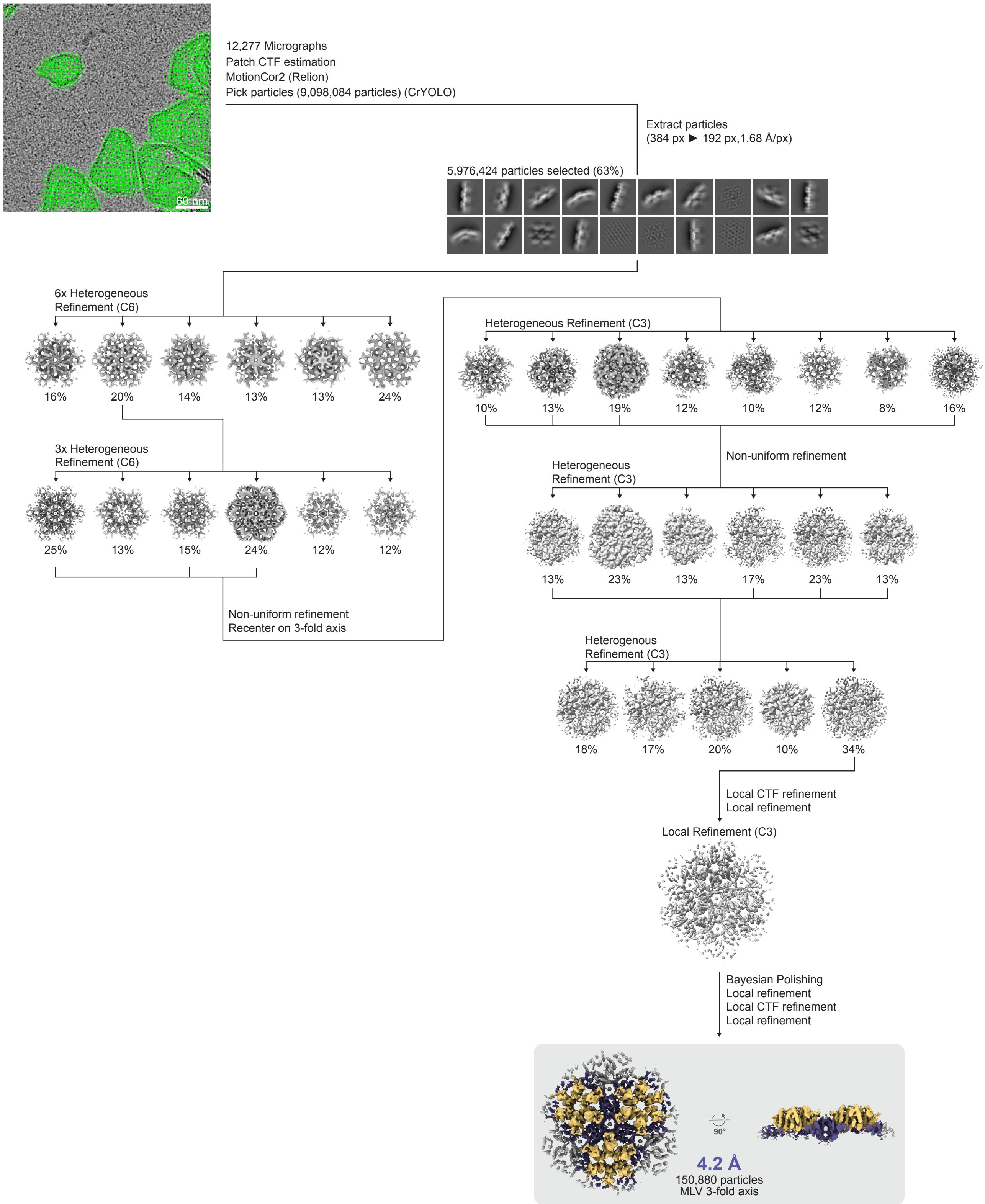

Supplementary Figure 5. Cryo-EM data processing workflow for MLV hexamer 3-fold interface structure from CLPs. Particle picking across the CLP surface was automated in crYOLO. High-quality particle picks were selected in 2D classification, and hexamer positions were selected by heterogeneous refinement. Hexamer positions were recentred on the adjacent 3-fold axes and further classified. In the final reconstruction, the CA_{NTD} and CA_{CTD} of central capsomers are shown in yellow and purple, respectively.

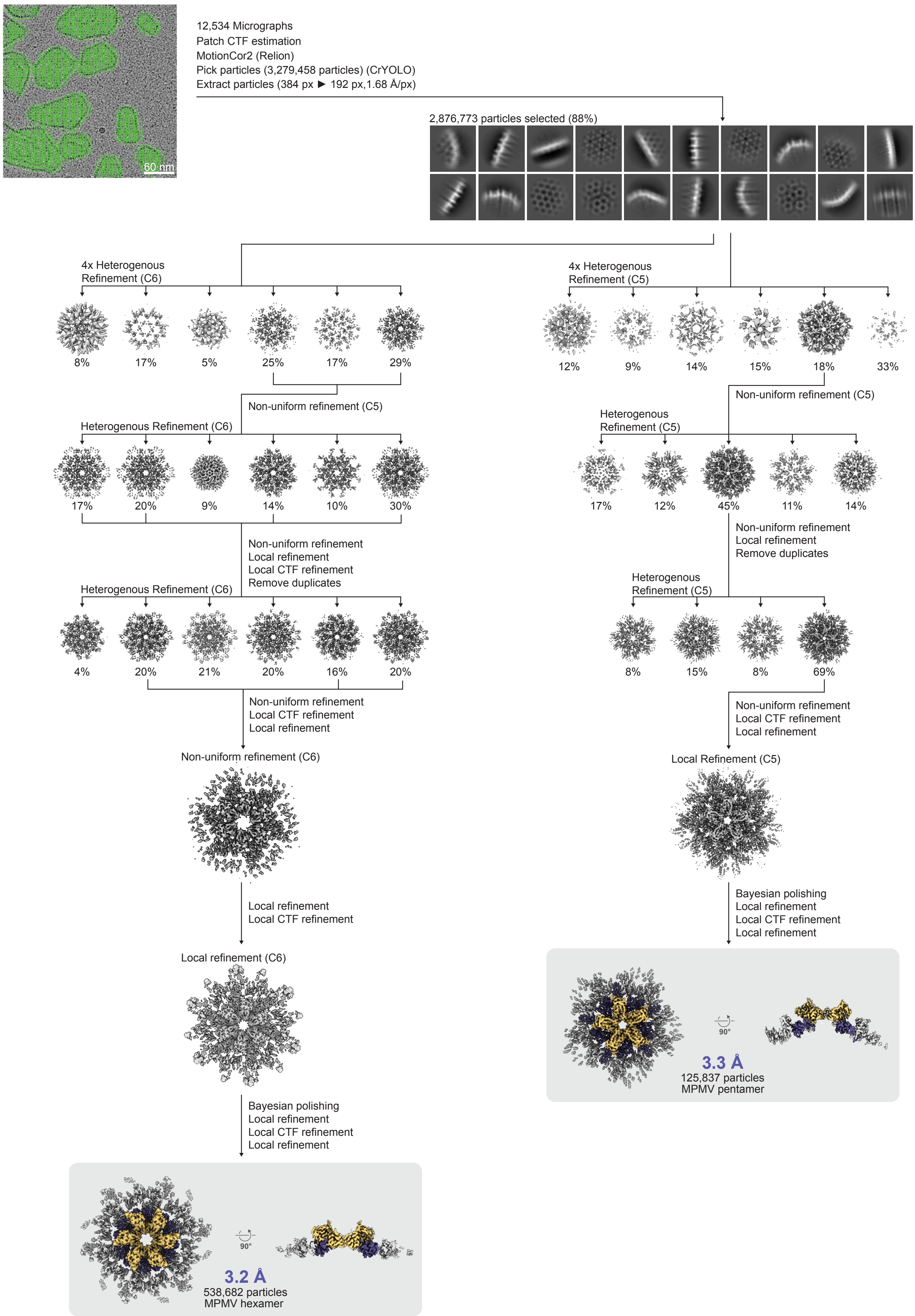

Supplementary Figure 6. Cryo-EM data processing workflow for MPMV hexamer and pentamer structures from CLPs. Particle picking across the CLP surface was automated in crYOLO. High-quality particle picks were selected in 2D classification, and hexamer and pentamer positions were sorted by heterogeneous refinement. In the final reconstruction, the CA_{NTD} and CA_{CTD} of central capsomers are shown in yellow and purple, respectively.

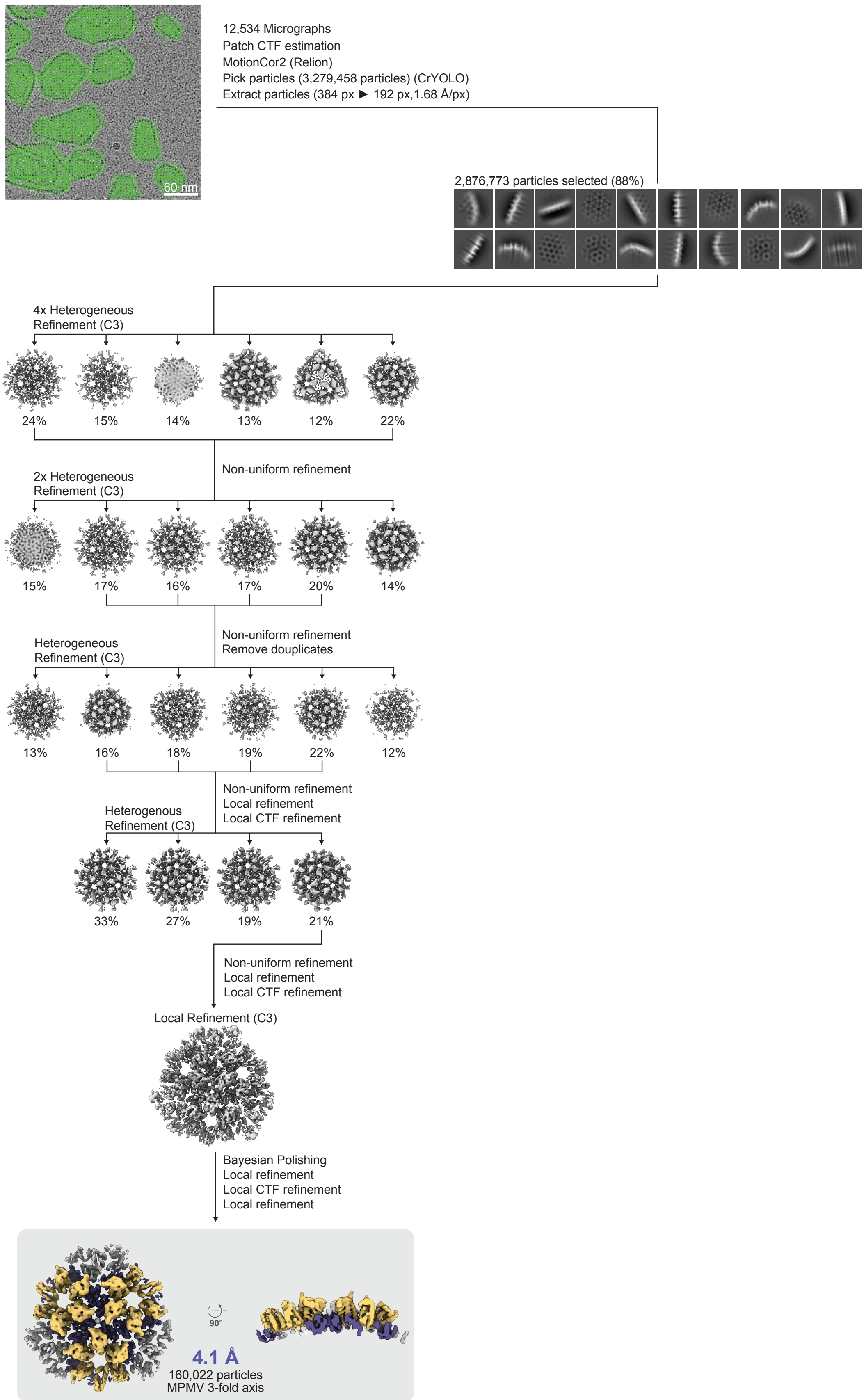

Supplementary Figure 7. Cryo-EM data processing workflow for MPMV hexamer 3-fold interface structure from CLPs. Particle picking across the CLP surface was automated in crYOLO. High-quality particle picks were selected in 2D classification, and 3-fold axis positions were selected by heterogeneous refinement. In the final reconstruction, the CA_{NTD} and CA_{CTD} of central capsomers are shown in yellow and purple, respectively.

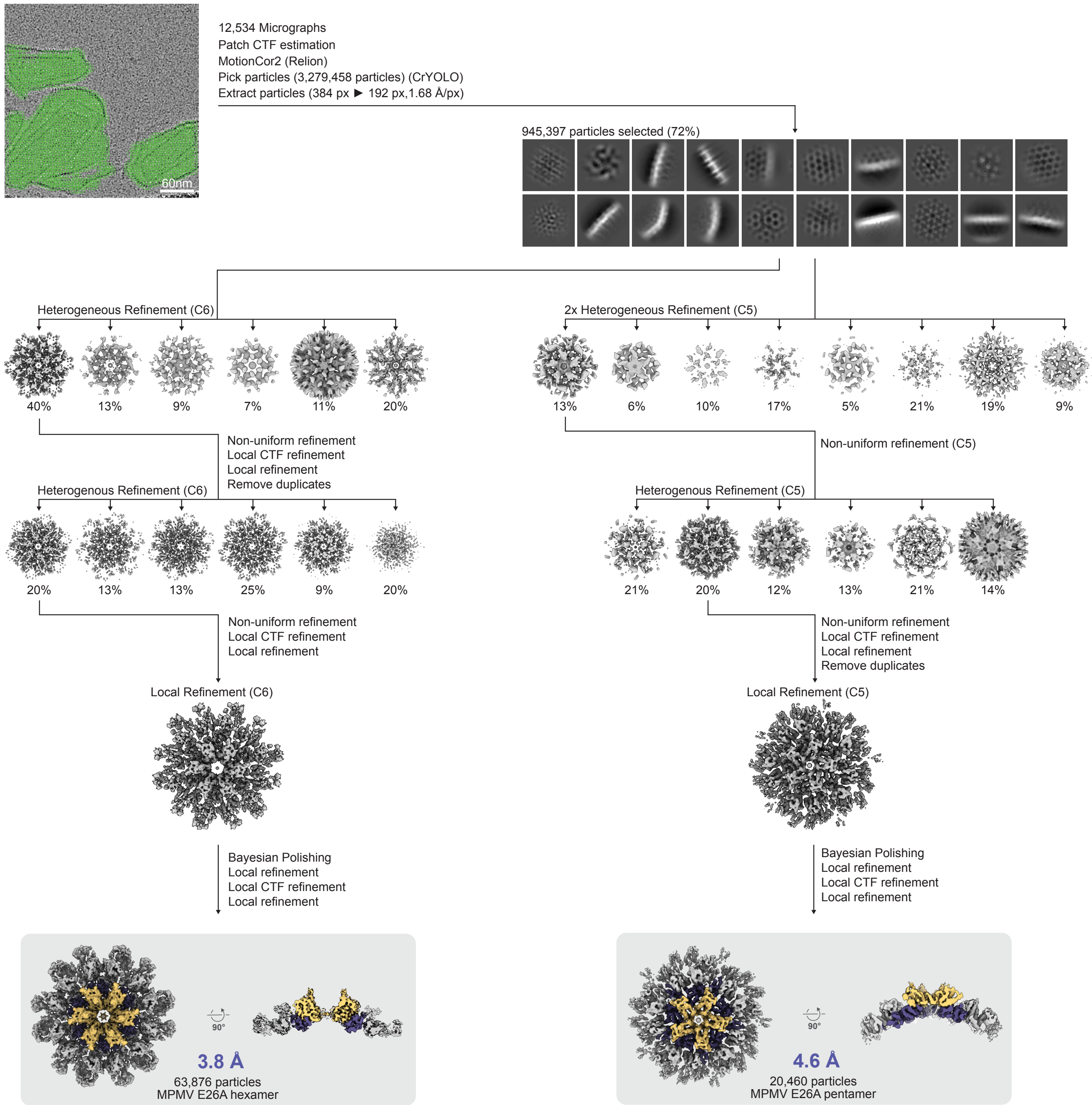

Supplementary Figure 8. Cryo-EM data processing workflow for MPMV E26A hexamer and pentamer structures from CLPs. Particle picking across the CLP surface was automated in crYOLO. High-quality particle picks were selected in 2D classification, and hexamer and pentamer positions were sorted by heterogeneous refinement. In the final reconstruction, the CA_{NTD} and CA_{CTD} of central capsomers are shown in yellow and purple, respectively.

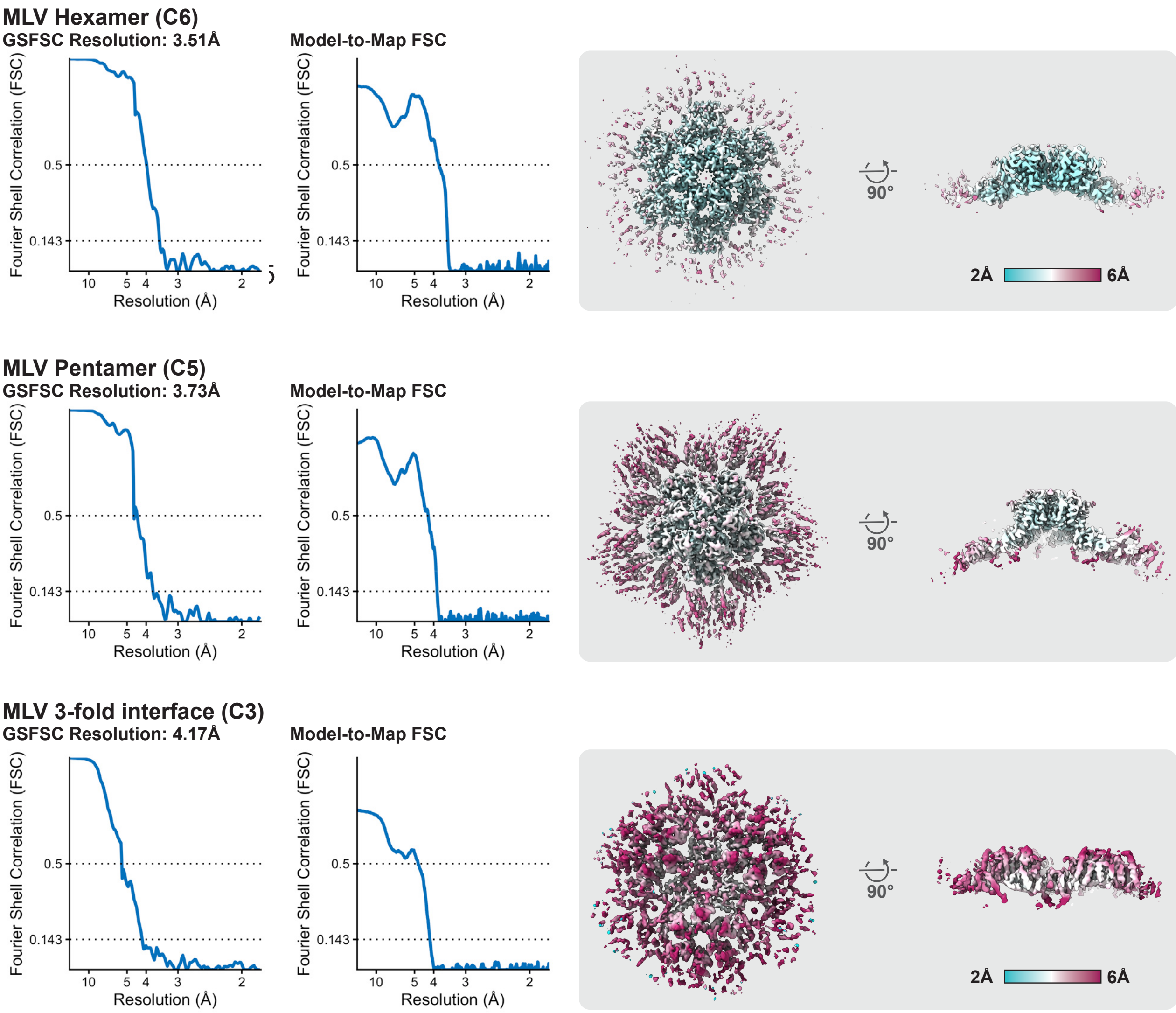

Supplementary Figure 9. FSC plots and local resolution maps for MLV single-particle CryoEM reconstructions. FSC plots and local resolution maps for all final reconstructions were computed in cryoSPARC. Model-to-Map FSC plots were computed with Phenix.

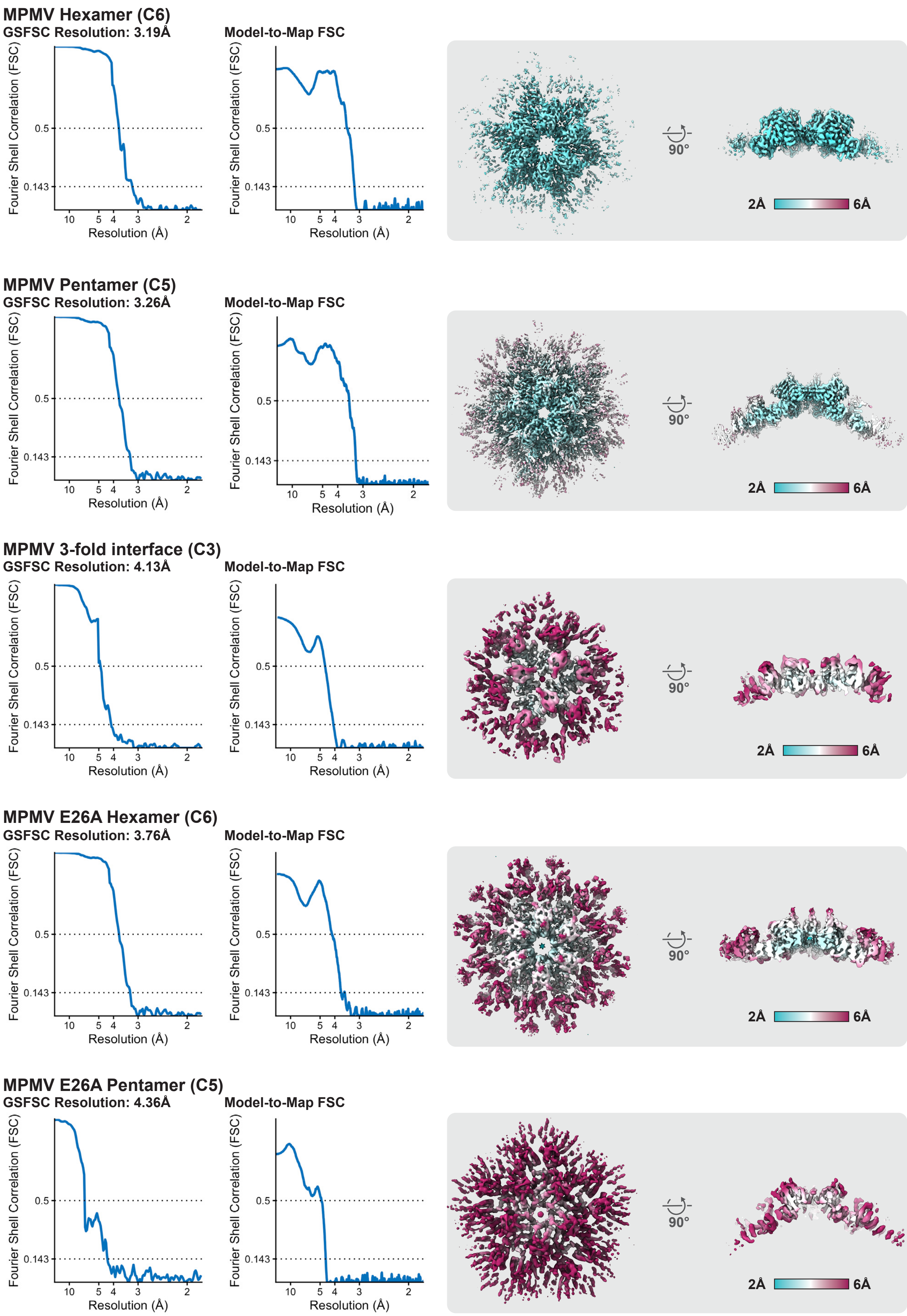

Supplementary Figure 10. FSC plots and local resolution maps for MPMV wild-type and E26A single-particle CryoEM reconstructions. FSC plots and local resolution maps for all final reconstructions were computed in cryoSPARC. Model-to-Map FSC plots were computed with Phenix.
