# Supplementary material for: Retroviruses use different IP6 binding mechanisms to alter the properties of their capsids
Source: Nat Commun. 2026 Aug 21;17:10005. doi: 10.1038/s41467-026-76510-7 (PMC13590620; doi:10.1038/s41467-026-76510-7)
Supplement: Supplementary file 2 [file 41467_2026_76510_MOESM2_ESM.pdf]

nature portfolio

Corresponding author(s): Leo C. James
Last updated by author(s): Jun 8, 2026

Reporting Summary

Nature Portfolio wishes to improve the reproducibility of the work that we publish. This form provides structure for consistency and transparency in reporting. For further information on Nature Portfolio policies, see our Editorial Policies and the Editorial Policy Checklist.

Statistics

For all statistical analyses, confirm that the following items are present in the figure legend, table legend, main text, or Methods section.

n/a	Confirmed	
☐	☒	The exact sample size (*n*) for each experimental group/condition, given as a discrete number and unit of measurement
☐	☒	A statement on whether measurements were taken from distinct samples or whether the same sample was measured repeatedly
☐	☒	The statistical test(s) used AND whether they are one- or two-sided<br>*Only common tests should be described solely by name; describe more complex techniques in the Methods section.*
☒	☐	A description of all covariates tested
☐	☒	A description of any assumptions or corrections, such as tests of normality and adjustment for multiple comparisons
☐	☒	A full description of the statistical parameters including central tendency (e.g. means) or other basic estimates (e.g. regression coefficient) AND variation (e.g. standard deviation) or associated estimates of uncertainty (e.g. confidence intervals)
☐	☒	For null hypothesis testing, the test statistic (e.g. *F*, *t*, *r*) with confidence intervals, effect sizes, degrees of freedom and *P* value noted<br>*Give P values as exact values whenever suitable.*
☒	☐	For Bayesian analysis, information on the choice of priors and Markov chain Monte Carlo settings
☒	☐	For hierarchical and complex designs, identification of the appropriate level for tests and full reporting of outcomes
☒	☐	Estimates of effect sizes (e.g. Cohen's *d*, Pearson's *r*), indicating how they were calculated

Our web collection on statistics for biologists contains articles on many of the points above.

Software and code

Policy information about availability of computer code

Data collection	Thermofisher EPU was used for cryoEM data collection, Jess Simple Western System with Compass software was used for automated Western Blotting.
Data analysis	IncuCyte S3 Live-Cell Analysis System was used for infection assay data collection and analysis. Jess Simple Western System with Compass software for simple western was used for Western Blot quantification. GraphPad PRISM-11 software was used for data analysis and presentation.<br>Relion-5.0, MotionCorr2, crYOLO v1.9.9, CryoSPARC v4.4.1 were used for single particle data analysis. Coot 0.9.8.95, and phenix-1.21.2 were used for model building and refinement. ChimeraX-1.9 was used for data presentation

For manuscripts utilizing custom algorithms or software that are central to the research but not yet described in published literature, software must be made available to editors and reviewers. We strongly encourage code deposition in a community repository (e.g. GitHub). See the Nature Portfolio guidelines for submitting code & software for further information.

Data

Policy information about availability of data

All manuscripts must include a data availability statement. This statement should provide the following information, where applicable:

- Accession codes, unique identifiers, or web links for publicly available datasets
- A description of any restrictions on data availability
- For clinical datasets or third party data, please ensure that the statement adheres to our policy

The cryo-electron microscopy density maps have been deposited in the Electron Microscopy Data Bank (EMDB) under accession codes EMD-56387 [https://www.ebi.ac.uk/emdb/EMD-56387] (MoMLV 3FA), EMD-56388 [https://www.ebi.ac.uk/emdb/EMD-56388] (MoMLV Hex), EMD-56389 [https://www.ebi.ac.uk/emdb/EMD-56389] (MoMLV Pent), EMD-56390 [https://www.ebi.ac.uk/emdb/EMD-56390] (MPMV 3FA), EMD-56394 [https://www.ebi.ac.uk/emdb/EMD-56394] (MPMV Hex), EMD-56393 [https://www.ebi.ac.uk/emdb/EMD-56393] (MPMV Pent), EMD-56392 [https://www.ebi.ac.uk/emdb/EMD-56392] (MPMV E26A Hex), EMD-56391 [https://www.ebi.ac.uk/emdb/EMD-56391] (MPMV E26A Pent). The corresponding molecular models have been deposited in the Protein Data Bank (PDB) under accession codes 9TX4 [https://doi.org/10.2210/pdb9tx4/pdb] (MoMLV 3FA), 9TX5 [https://doi.org/10.2210/pdb9tx5/pdb] (MoMLV Hex), 9TX6 [https://doi.org/10.2210/pdb9tx6/pdb] (MoMLV Pent), 9TX7 [https://doi.org/10.2210/pdb9tx7/pdb] (MPMV 3FA), 9TXB [https://doi.org/10.2210/pdb9txb/pdb] (MPMV Hex), 9TXA [https://doi.org/10.2210/pdb9txa/pdb] (MPMV Pent), 9TX9 [https://doi.org/10.2210/pdb9tx9/pdb] (MoMLV E26A Hex), 9TX8 [https://doi.org/10.2210/pdb9tx8/pdb] (MoMLV E26A Pent). Source data are provided with this paper.

Research involving human participants, their data, or biological material

Policy information about studies with human participants or human data. See also policy information about sex, gender (identity/presentation), and sexual orientation and race, ethnicity and racism.

Reporting on sex and gender	NA
Reporting on race, ethnicity, or other socially relevant groupings	NA
Population characteristics	NA
Recruitment	NA
Ethics oversight	NA

Note that full information on the approval of the study protocol must also be provided in the manuscript.

Field-specific reporting

Please select the one below that is the best fit for your research. If you are not sure, read the appropriate sections before making your selection.

☒ Life sciences ☐ Behavioural & social sciences ☐ Ecological, evolutionary & environmental sciences

For a reference copy of the document with all sections, see nature.com/documents/nr-reporting-summary-flat.pdf

Life sciences study design

All studies must disclose on these points even when the disclosure is negative.

Sample size	NA
Data exclusions	For CryoEM single particle data processing, particles were subjected to classification leading to exclusion of some images. This is a standard processing step and detailed further in the Methods section.<br>For infection data, plateauing data points were excluded for fitting in the linear range as a standard processing step that is required for correct data representation. Data points within the first 5 minutes of assembly reactions are not shown to excluded the time span in which buffer addition created an inhomogenous solution that falsely increased values by diffraction at phase borders.
Replication	Data were collected with technical replicates; experiments were repeated three times to produce biological replicates, and all replicates were successful.
Randomization	NA
Blinding	NA

Reporting for specific materials, systems and methods

We require information from authors about some types of materials, experimental systems and methods used in many studies. Here, indicate whether each material, system or method listed is relevant to your study. If you are not sure if a list item applies to your research, read the appropriate section before selecting a response.

Materials & experimental systems

n/a	Involved in the study	
☐	☒	Antibodies
☐	☒	Eukaryotic cell lines
☒	☐	Palaeontology and archaeology
☒	☐	Animals and other organisms
☒	☐	Clinical data
☒	☐	Dual use research of concern
☒	☐	Plants

Methods

n/a	Involved in the study	
☒	☐	ChIP-seq
☒	☐	Flow cytometry
☒	☐	MRI-based neuroimaging

Antibodies

Antibodies used	Polyclonal antibody against MPMV CA (non-commerical, raised lab-internally)<br>Polyclonal antibody against Mo-MLV CA (non-commerical, raised lab-internally)
Validation	Antibodies were validated by ELISA using purified MPMV CA or Mo-MLV protein respectively.

Eukaryotic cell lines

Policy information about cell lines and Sex and Gender in Research

Cell line source(s)	293T CRL-3216 cells from ATCC
Authentication	293T CRL-3216 cells were authenticated by by STR profiling.
Mycoplasma contamination	ATCC tested as mycoplasma-free
Commonly misidentified lines<br>(See ICLAC register)	No No commonly misidentified cell lines were used.

Plants

Seed stocks	NA
Novel plant genotypes	NA
Authentication	NA
