# Supplementary material for: Retroviruses use different IP6 binding mechanisms to alter the properties of their capsids
Source: Nat Commun. 2026 Aug 21;17:10005. doi: 10.1038/s41467-026-76510-7 (PMC13590620; doi:10.1038/s41467-026-76510-7)
Supplement: Supplementary file 3 [file 41467_2026_76510_MOESM3_ESM.pdf]

nature portfolio

Peer Review File

Retroviruses use different IP6 binding mechanisms to alter the properties of their capsids

Corresponding Author: Dr Leo James

This file contains all reviewer reports in order by version, followed by all author rebuttals in order by version.

Version 0:

Reviewer comments:

Reviewer #1

(Remarks to the Author)
This is review of the manuscript "Retroviruses use different IP6 binding mechanisms to alter the properties of their capsids" by Klarhof et al., The manuscript is a highly significant and well-executed study that fundamentally expands our understanding of retroviral capsid assembly and maturation. By investigating the beta-retrovirus Mason-Pfizer Monkey Virus (MPMV) and the gamma-retrovirus Murine Leukaemia Virus (MLV), the authors convincingly demonstrate that the use of inositol hexakisphosphate (IP6) as a capsid co-factor is a broadly conserved retroviral strategy, rather than a lentivirus-specific quirk.
Crucially, the manuscript reveals an entirely novel structural paradigm: whereas HIV-1 binds IP6 within the central pore of individual capsomers, MPMV and MLV coordinate IP6 at the 3-fold symmetry axis between capsomers. The integration of high-resolution cryo-EM, in vitro assembly kinetics, and cellular infectivity assays using IP-knockout cell lines provides a robust, multi-disciplinary foundation for the authors' claims.

Strengths and Significance

Paradigm-Shifting Structural Discoveries: The field has heavily relied on the HIV-1 model, where IP6 is coordinated by rings of charged residues (e.g., R18 and K25) within the central capsomer pore. By solving the structures of MPMV and MLV core-like particles (CLPs), the authors identified a previously unknown IP6 binding site at the inter-capsomer 3-fold axis.

Clarification of MLV Literature: The authors directly address and resolve recent conflicting literature regarding MLV. A recent study by Biswas et al. (2024) suggested that the R3 residue in the MLV capsomer pore was the likely binding site for IP6. This manuscript uses definitive structural evidence to show that IP6 actually binds K193 and K218 at the 3-fold axis, clarifying that while R3 is essential for infectivity, it is not the primary IP6 coordination site.

Nuanced Biological Phenotyping: The study elegantly connects structural differences to viral life-cycle dependencies. It demonstrates that the inter-capsomer binding in MPMV and MLV makes them less dependent on IP6 during virion production (unlike HIV-1, which actively packages IP6), but highly dependent on scavenging IP6/IP5 to maintain capsid stability upon entering target cells.

High-Quality Cryo-EM Pipeline: The structural pipeline utilizes state-of-the-art data collection (Titan Krios, Falcon 4) and rigorous processing workflows (cryoSPARC, RELION, crYOLO), achieving highly credible resolutions of 3.2–3.9 Å for capsomers and ~4.1-4.2 Å for the more challenging 3-fold interface reconstructions.

Critique and Recommendations for the Authors
While the manuscript is excellent, addressing the following points could further strengthen the narrative and impact:
1. The "Black Box" of the MLV R3 Residue
Observation: You accurately show that an R3A mutation delays MLV assembly and abolishes infectivity, yet the cryo-EM maps prove R3 does not bind IP6.
Recommendation: Given the emphasis placed on R3 by Biswas et al. (2024) , it would be beneficial to expand the discussion slightly on what R3 might actually be doing. Does it play a role in host-factor recruitment, core condensation, or pore-gating for nucleotide import (similar to the dynamic pores in HIV-1)?

2. IP3/IP4 Coordination Hypothesis in MPMV
Observation: A fascinating biological difference is highlighted: MPMV can utilize smaller inositol phosphates (IP3, IP4) for assembly, whereas MLV cannot. You hypothesize that MPMV's dual-lysine rings (K87 and K203 on different planes) might coordinate two IP3/IP4 molecules simultaneously, whereas MLV's single planar ring cannot.
Recommendation: This is a compelling hypothesis. While solving another structure with IP3 might be beyond the scope of this paper, performing simple in silico docking or electrostatic modelling of two IP3 molecules into the MPMV 3-fold axis could provide theoretical support for this claim and enrich the structural discussion.

3. Comparison to Previous MLV Maps
Observation: Qu et al. (2018) previously published the structure and architecture of MLV capsids, but did not identify IP6 at the 3-fold axis.
Recommendation: It would be helpful to briefly clarify why IP6 was visible here but not in earlier studies. Was it strictly due to the addition of 2.5 mM IP6 during your in vitro assembly, whereas previous studies lacked supplemented IP6? Clarifying this reinforces the absolute necessity of the metabolite for this structural feature.

4. Contextualizing K25 in MPMV
Observation: You beautifully demonstrate that MPMV K25 forms a salt bridge with E26 rather than binding IP6, and that the K25A/E26A double mutant can paradoxically assemble better than WT in vitro but is non-infectious.
Recommendation: Discuss briefly why this salt bridge is essential for infectivity if it isn't strictly required for assembly. Could it be required for maintaining appropriate core flexibility, uncoating kinetics, or reverse transcription?

This is a highly rigorous manuscript that provides a crucial missing link in retrovirology, proving that nature found multiple structural solutions to utilize the same host metabolite (IP6) for viral capsid stabilization.

Reviewer #2

(Remarks to the Author)
Klarhof et al describe and compare the differential mechanism of IP6 binding to capsid lattices amongst 3 different retroviruses (lenti-, beta- and gamma-retroviruses), using a combination of biochemical, virological and structural approaches. The authors show that homologous residues in MPMV and MLV that may mimic the role of IP6 binding in HIV capsid core, fail to affect capsid core assembly. Instead using cryo-EM analysis, the authors then show density potentially arising from IP6 at the pore-like region of the 3-fold axis of capsomeres, thereby discovering a new interface for IP6 binding. Substituting residues within the pore-like region in the 3-fold axis affects infectivity indicating a strong role for these residues in maintaining optimal capsid core stability. Contrary to HIV, in which IP6 availability is critical in producer cells, in MPMV and MLV, IP6/IP5 availability appears to be more important in target cells. Additionally, MPMV and not MLV cores can be stabilized through availability of other forms like IP4 and IP3. Overall, the results of this manuscript are exciting as it suggests subtle differences with respect to capsid uncoating dynamics amongst retroviruses, potentially resulting in subtle differences with respect to functional genome delivery in the respective retroviruses. Understanding these differences will be key in development of better inhibitors for HIV-1 and more broadly understanding the cellular pathogenesis of different retroviral infections. However, the authors need to clarify several important aspects before the manuscript is suitable for publication.

Major comments-
1. For MPMV, although the data suggests disruption of IP5 and IP6 production in both producer and target cells influences infectivity (which the authors don't describe with respect to quantification), there is a clear defect in proteolytic processing of CA in IMPK cells (Fig S2D,F). Do the authors control for infectivity by measuring RT or cleaved CA? I suspect the apparent defect in IMPK producer/target cells is dictated in large part due to defective CA processing. If this is true, then there is potentially a role for residues important for IP5/IP6 in CA assembly in producer cells for MPMV and MLV, like HIV. How do the authors reconcile this defective processing defect with respect to CA assembly?
2. I assume the antibody used for MLV CA does not detect CA within Gag since those blots show only a CA band in western blots (Fig S3C). So, for MLV it isn't clear yet whether there is a similar issue of improper Gag processing. However, it appears in MLV, K193A and K193A/K218A have similar defects in processing. The data therefore cannot be attributed to IP6 interaction disruption alone. The western blots for MLV CA are confusing. How are you not seeing processed CA in virus particles for these mutants but seeing them in producer cells? Do these mutants have budding defects? (Fig S3A). In MPMV you see processed and unprocessed CA in cells and mostly processed CA in virus, which is what one would expect. But this does not seem to be the case for MLV.
3. Please revisit line 270-272 as the IMPK part is inconsistent with the subsequent sentences. "Consistent with published data, we did not observe any reduction in viral production of WT MLV in IPPK KOs and only a modest decrease in IPMK KOs overexpressing MINPP1 (Fig. S3B,C)". To me this is not a modest decrease, but rather a significant decrease in virus production in IPMK cells. Especially when compared with MPMV WT, where the magnitude of virus production is not impacted as much depending on cell type. This implies IP5 and/or IP6 may be important for assembly in producer cells too, in the case of MLV.
Minor comments-
There are several instances of mislabeling, which should be carefully rechecked before resubmission.
1. Fig 3A if white is negative/neutral and blue is positive what is red on the surface maps?
2. Fig 4 legends are mixed up between panels B and D.
3. Fig 6 panels A and B do not show white empty circles denoting individual data points. For sake of consistency please include those. Also, Fig 6 panel legends have been mislabeled.

4. In the Methods section, please describe further how specimens prepared for vitrification? Is IP6 supplemented during vitrification for cryo-EM or is it present during protein purification? Currently, there is no mention of IP6 being supplemented.
5. Sup Fig3A. There is no Pr78 in MLV, please label with the correct precursor product for MLV.
6. Sup Fig3B. shouldn't it be MLV and not MPMV in figure legend?
7. Sup Fig3C, right most panel, are the p30-like bands an artifact in the beta-actin blot? If so, the labeling is confusing. The figure would be easier to interpret if you mark the artifact with an asterisk and move p30 to the other blots which are probed using anti-CA antibody.

Reviewer #3

(Remarks to the Author)
IP6 is known to play a critical role in the assembly of both immature and mature HIV-1 Gag lattices. However, the capsid residues that coordinate IP6 in HIV-1 are not conserved across retroviruses. In this well-executed study, James and colleagues demonstrate that the betaretrovirus MPMV and the gammaretrovirus MLV also utilize IP6 (and potentially other inositol phosphates, such as IP5). Strikingly, these viruses coordinate the metabolite at the threefold axis between capsomers, rather than at the sixfold axis within individual capsomers as observed in HIV-1.

These findings reveal an intriguing example of convergent evolution and suggest that the use of polyanionic metabolites may be a general feature of orthoretroviruses. The study further shows that IP6 dependence is not intrinsically hardwired into capsid assembly: mutants lacking the positively charged residues required for IP6 coordination lose their dependence on the metabolite. This indicates that individual viruses have evolved to exploit a dissociable cofactor to confer specific functional advantages.

I have only minor suggestions. The figures are well designed and highly effective; however, the writing is occasionally sloppy, and the manuscript would benefit from careful proofreading.

Line 240: "Using single particle reconstruction, we reconstructed…"
Line 335: "structural differences with infectivity data highlights several important differences"
Line 354: "spuma-viruses don't require", Spumaviruses do not require?
Lines 402, 410: "50mM TRIS pH=8.0", Tris-HCl, pH 8.0?
Table 2 title should be "Model refinement.."

Lines 191-193: I am not sure if the data in Fig 3C support synergistic action.

Version 1:

Reviewer comments:

Reviewer #1

(Remarks to the Author)
I have found the response satisfactory

Reviewer #2

(Remarks to the Author)
Authors have addressed all of my concerns.

Open Access This Peer Review File is licensed under a Creative Commons Attribution 4.0 International License, which permits use, sharing, adaptation, distribution and reproduction in any medium or format, as long as you give appropriate credit to the original author(s) and the source, provide a link to the Creative Commons license, and indicate if changes were made.
In cases where reviewers are anonymous, credit should be given to 'Anonymous Referee' and the source.
The images or other third party material in this Peer Review File are included in the article's Creative Commons license, unless indicated otherwise in a credit line to the material. If material is not included in the article's Creative Commons license and your intended use is not permitted by statutory regulation or exceeds the permitted use, you will need to obtain permission directly from the copyright holder.
To view a copy of this license, visit https://creativecommons.org/licenses/by/4.0/

Response to Reviewers

Reviewer #1 (Remarks to the Author):

Critique and Recommendations for the Authors
While the manuscript is excellent, addressing the following points could further strengthen the narrative and impact:
Q1. The "Black Box" of the MLV R3 Residue
Observation: You accurately show that an R3A mutation delays MLV assembly and abolishes infectivity, yet the cryo-EM maps prove R3 does not bind IP6.
Recommendation: Given the emphasis placed on R3 by Biswas et al. (2024) , it would be beneficial to expand the discussion slightly on what R3 might actually be doing. Does it play a role in host-factor recruitment, core condensation, or pore-gating for nucleotide import (similar to the dynamic pores in HIV-1)?

A1. We have added the following to the discussion:
Line 329: "This finding contrasts with a recent study suggesting that MLV residue R3 coordinates IP6 for capsid assembly in the capsomer pore (Biswas et al., 2024). However, our results show that R3A can assemble in the presence of IP6 and that while R3A is non-infectious, MLV virions retain some infectivity when produced in, or added to, IP6-depleted cells. The severity of the R3A infection phenotype therefore suggests that it has a critical function distinct from IP6 binding. This may be the binding of a different cofactor or the import of dNTPs for reverse transcription, analogous to the HIV 1 pore (Jacques et al., 2016)."

Q2. IP3/IP4 Coordination Hypothesis in MPMV
Observation: A fascinating biological difference is highlighted: MPMV can utilize smaller inositol phosphates (IP3, IP4) for assembly, whereas MLV cannot. You hypothesize that MPMV's dual-lysine rings (K87 and K203 on different planes) might coordinate two IP3/IP4 molecules simultaneously, whereas MLV's single planar ring cannot.
Recommendation: This is a compelling hypothesis. While solving another structure with IP3 might be beyond the scope of this paper, performing simple in silico docking or electrostatic modelling of two IP3 molecules into the MPMV 3-fold axis could provide theoretical support for this claim and enrich the structural discussion.

A2. Thank you for this suggestion. We have docked two IP3 molecules into the MPMV 3FA structural model and made a comparative figure (S1C).

Q3. Comparison to Previous MLV Maps
Observation: Qu et al. (2018) previously published the structure and architecture of MLV capsids, but did not identify IP6 at the 3-fold axis.
Recommendation: It would be helpful to briefly clarify why IP6 was visible here but not in earlier studies. Was it strictly due to the addition of 2.5 mM IP6 during your in vitro assembly, whereas previous studies lacked supplemented IP6? Clarifying this reinforces the absolute necessity of the metabolite for this structural feature.

A3. We have included additional sentences in the results and the discussion:
Line 249: "This density was less prominent in previous studies, likely because the preparations were not supplemented with IP6 and thus limited by the molecules packaged within the virions (Qu et al., 2018)."
Line 359: "The absence of IP6 packaging may explain why density for the metabolite is weak at the 3-fold axis in capsid structures solved from virions (Qu et al., 2018), while MLV's sensitivity to IP6 levels in target cells suggests binding to the capsid also takes place post-entry."

Q4. Contextualizing K25 in MPMV
Observation: You beautifully demonstrate that MPMV K25 forms a salt bridge with E26 rather than binding IP6, and that the K25A/E26A double mutant can paradoxically assemble better than WT in vitro but is non-infectious.

Recommendation: Discuss briefly why this salt bridge is essential for infectivity if it isn't strictly required for assembly. Could it be required for maintaining appropriate core flexibility, uncoating kinetics, or reverse transcription?

A4. We agree that it could be required for any of these functions and have followed the suggestion to mention this:
Line 164: "It may play a role in maintaining capsid stability, or possibly K25 is used to mediate dNTP import, similar to R18 and K25 in HIV-1 (Jacques et al., 2016), and interaction with E26 occurs in the absence of nucleotides to prevent charge repulsion.'

Q5. This is a highly rigorous manuscript that provides a crucial missing link in retrovirology, proving that nature found multiple structural solutions to utilize the same host metabolite (IP6) for viral capsid stabilisation.

A5. We thank the reviewer for this and their other positive comments and useful suggestions.

Reviewer #2 (Remarks to the Author):

We thank the reviewer for their thorough review of our data and for their comments. We believe we have addressed the points of criticism below: In multiple places, we have additionally included explanations and specified the wording that might have contributed to a different interpretation of the presented data.

Major comments-
Q1. For MPMV, although the data suggests disruption of IP5 and IP6 production in both producer and target cells influences infectivity (which the authors don't describe with respect to quantification), there is a clear defect in proteolytic processing of CA in IMPK cells (Fig S2D,F). Do the authors control for infectivity by measuring RT or cleaved CA? I suspect the apparent defect in IMPK producer/target cells is dictated in large part due to defective CA processing. If this is true, then there is potentially a role for residues important for IP5/IP6 in CA assembly in producer cells for MPMV and MLV, like HIV. How do the authors reconcile this defective processing defect with respect to CA assembly?

A1. We control for infectivity using quantification of cleaved CA and have added the following to the methods text to clarify this:
Line 457: "MPMV preparations were quantified based on CA concentration using automated western blotting on Jess, as detailed in the methods section below. The bands were quantified by densitometry with purified CA protein as a standard."
We agree there is a detectable uncleaved MPMV CA band in IPMK knockout cell lysate, as well as somewhat lower overall Gag expression. However, for the matched MPMV virion samples (left blot), almost complete Gag processing is observed. Hence, we conclude that for packaged Gag, processing does occur.

Q2. I assume the antibody used for MLV CA does not detect CA within Gag since those blots show only a CA band in western blots (Fig S3C). So, for MLV it isn't clear yet whether there is a similar issue of improper Gag processing. However, it appears in MLV, K193A and K193A/K218A have similar defects in processing. The data therefore cannot be attributed to IP6 interaction disruption alone. The western blots for MLV CA are confusing. How are you not seeing processed CA in virus particles for these mutants but seeing them in producer cells? Do these mutants have budding defects? (Fig S3A). In MPMV you see processed and unprocessed CA in cells and mostly processed CA in virus, which is what one would expect. But this does not seem to be the case for MLV.

A2. We apologise for the confusion, which is the result of our use of two different detection methods (traditional WB and Jess) and two different antibodies, one of which only detects cleaved CA. We have now repeated the blots so that they are both traditional WBs. As can be seen in the new Fig 3S, we can detect both full-length Gag and cleaved CA, although in some samples processing is either complete or Gag levels are so low that it is sometimes difficult to see. The mutants K193A and K193A/K218A have lower Gag expression in producer cells, and we see a corresponding reduction in viral production. To take into account differences in virion production, we normalise our input virus based on CA levels for further

infection studies. It also looks like there may be less efficient processing of Gag in these mutants. To address that processing might impact infectivity, we have amended the relevant paragraph in the results chapter accordingly:
Line 265: "To assess whether the 3-fold axis IP6 binding site is important for MLV infectivity, we mutated K193 and K218 individually and together (Fig. S3A). Each mutant significantly reduced MLV infectivity, and a combined effect was observed when both K193A and K218A were mutated, reminiscent of that observed with the MPMV double mutant K87A/K203A (compare Fig. 3E & Fig. 5D). Mutants involving K193A also had a production defect and reduced processing compared to WT, likely contributing to their infectivity defect. The K218A mutation had a clear infectivity defect with minimal effects on production and processing. We hence conclude that IP6 binding is important for MLV infectivity and used the K218A mutant for further experiments."

Experiments comparing the infectivity of viruses produced or added to IP6-depleted cells also give us confidence that the infectivity defect we see in K218A is, at least in part, IP6-dependent. This is because K218A loses much of its sensitivity to cellular IP6 levels, compared to WT MLV.

Q3. Please revisit line 270-272 as the IMPK part is inconsistent with the subsequent sentences. "Consistent with published data, we did not observe any reduction in viral production of WT MLV in IPPK KOs and only a modest decrease in IPMK KOs overexpressing MINPP1 (Fig. S3B,C)". To me this is not a modest decrease, but rather a significant decrease in virus production in IPMK cells. Especially when compared with MPMV WT, where the magnitude of virus production is not impacted as much depending on cell type. This implies IP5 and/or IP6 may be important for assembly in producer cells too, in the case of MLV.

A3. We have revised the text in the indicated paragraph to clarify the type of defect observed in IPMK KO cells:
Line 279: "Consistent with published data, we did not observe any reduction in viral production of WT MLV in IPPK KOs (Fig. S3B,C). Fewer virions were produced in IPMK KO cells overexpressing MINPP1 and this coincided with reduced overall CA expression in the cell lysate (Fig. S3B,C)."

Minor comments-
There are several instances of mislabeling, which should be carefully rechecked before resubmission.
1. Fig 3A if white is negative/neutral and blue is positive, what is red on the surface maps?
Thank you for pointing out this mistake in the figure legend. We have corrected it.
2. Fig 4 legends are mixed up between panels B and D.
We have corrected the legend to match the panels.
3. Fig 6 panels A and B do not show white empty circles denoting individual data points. For sake of consistency please include those. Also, Fig 6 panel legends have been mislabeled.
The proposed symbols have been added to the graphs. The figure legend has also been rearranged to match the updated panel labels.
4. In the Methods section, please describe further how specimens prepared for vitrification? Is IP6 supplemented during vitrification for cryo-EM or is it present during protein purification? Currently, there is no mention of IP6 being supplemented.
The section was changed to a more accurate wording: "Capsid proteins were pre-diluted to yield identical concentrations across all tested mutants. Assembly reactions were initiated by the addition of IP6 (or other indicated inositol phosphates) as part of a 10-fold concentrated buffer. Buffer addition adjusted the reaction conditions to 50mM TRIS pH=8.0, 2mM TCEP and IP concentrations as specified for each experiment."
5. Sup Fig3A. There is no Pr78 in MLV, please label with the correct precursor product for MLV.
Thank you for pointing out this mistake. It has been corrected.
6. Sup Fig3B. shouldn't it be MLV and not MPMV in figure legend?
Resolved; thank you!
7. Sup Fig3C, right most panel, are the p30-like bands an artifact in the beta-actin blot? If so, the labeling is confusing. The figure would be easier to interpret if you mark the artifact with an asterisk and move p30 to the other blots which are probed using anti-CA antibody.

We have incorporated your suggestion and described it accordingly in the figure legend: it is indeed an artefact of an incompletely stripped p30 blot.

Reviewer #3 (Remarks to the Author):

We thank the reviewer for their positive feedback. We have implemented the suggestion to go through the entire manuscript for another round of careful proofreading. The resulting changes are tracked as specified by the journal guidelines. The specific comments below have been addressed.

Line 240: "Using single particle reconstruction, we reconstructed..."
Changed to "Using a single particle cryoEM pipeline, we reconstructed..."
Line 335: "structural differences with infectivity data highlights several important differences"
"Linking these structural variations with infectivity data reveals several key differences between the viruses."
Line 354: "spuma-viruses don't require", Spumaviruses do not require?
We have made this change.
Lines 402, 410: "50mM TRIS pH=8.0", Tris-HCl, pH 8.0?
Adjusted to be consistently "Tris-HCl pH=8.0"
Table 2 title should be "Model refinement.."
We have corrected the title of Table 2. Thanks for spotting this.
Lines 191-193: I am not sure if the data in Fig 3C support synergistic action.
We have deleted the mention of synergy.
